# Genome-wide identification and molecular evolution of *Dof* transcription factors in *Cyperus esculentus*

**DOI:** 10.1186/s12864-024-10565-y

**Published:** 2024-07-03

**Authors:** Chun Fu, ZiHui Liao, Na Jiang, YaoJun Yang

**Affiliations:** 1 Key Laboratory of Sichuan Province for Bamboo Pests Control and Resource Development, Leshan Normal University, No. 778 Binhe Road, Shizhong District, Leshan, Sichuan 614000 China; 2 College of Life Science, Leshan Normal University, No. 778 Binhe Road, Shizhong District, Leshan, Sichuan 614000 China; 3 College of Tourism and Geographical Science, Leshan Normal University, No. 778 Binhe Road, Shizhong District, Leshan, Sichuan 614000 China

**Keywords:** Cyperus esculentus, Dof gene, Genome-wide identification, Transcription factors, Collinearity analysis

## Abstract

*Dof* transcription factor family in *Cyperus esculentus* genome was identified and analyzed using bioinformatics. The analysis results revealed that *C.esculentus* genome contains 29 *Dof* genes (*CesDof*), all of which are located in the nucleus according to subcellular localization prediction. CesDof proteinrs have a range of 124 to 512 amino acids, with most being basic proteins. Their secondary structure was mainly irregular curl. The promoter sequence of *CesDof* genes contains cis-acting elements that respond to light, drought, hormones, low temperature, and circadian rhythm. Codon preference analysis showed that *CesDof* genes' codon preference ends in T/A. Collinearity analysis revealed that *C.esculentus* had three pairs of collinear *CesDof* genes. Additionally, there were 15 pairs of collinear genes between *C.esculentus* and *Arabidopsis thaliana.* The genetic relationship between *C.esculentus* and *Rhynchospora pubera* was found to be the closest. Phylogenetic tree analysis revealed that 29 *CesDof* genes of *C.esculentus* can be classified into 4 subgroups. Additionally, 144 miRNAs were predicted to target these *CesDof* genes. Furthermore, protein interaction analysis indicated that 15 *Dof* proteins in *C.esculentus* had interactions. The qRT-PCR verification results of drought stress and salt stress treatment experiments showed that most *CesDof* genes were involved in drought stress and salt stress responses, and the gene expression trends under drought stress and salt stress conditions were consistent. These results lay a theoretical foundation for further studying the molecular functions of *Dof* gene family in *C.esculentus* and its molecular mechanisms in regulating the life activities of *C.esculentus.*

**Supplementary Information:**

The online version contains supplementary material available at 10.1186/s12864-024-10565-y.

## Introduction

*Cyperus esculentus* is a plant belonging to the genus Cyperus in the family Cyperaceae. It is a masculine plant that thrives in light, drought-resistant, waterlogging-resistant, barren, and saline-resistant conditions. The cake meal left after oil extraction can be used as concentrate feed for livestock and poultry. The stems and leaves of *C.esculentus* can also be used as high-quality fodder, which can be fed directly or made into hay in the sun [1]. *C.esculentus* is a non-food oilseed plant. Its oil has a protein and amino acid-rich composition, with an oil content of approximately 20% [2]. Biodiesel produced from the crude oil of *C.esculentus* seeds exhibits favourable fuel properties [3].

*Dof* (DNA binding with one finger) gene family is a plant-specific class of transcription factors that belong to the single zinc finger protein superfamily [4–6]. These factors typically consist of 200–400 amino acids and contain oligomerisation sites, nuclear localisation signals, and two functional structures [7, 8]. The protein has two main regions: the DNA-binding region located at the N-terminus, which is composed of 52 amino acids with a single zinc-finger conserved structural domain rich in cysteine residues, and the transcriptional regulatory region located at the C-terminus, which consists of a single zinc-finger conserved resultant region of a tryptophan [9, 10]. *Dof* gene family members recognize either the AAAG sequence-rich or the CTTTT sequence as the core recognition site. This site interacts with DNA or proteins to regulate gene expression and has a dual function [11, 12].

In 1993, scientists identified and reported the first *Dof* transcription factor from maize. Its main function is to participate in the regulation of plant photosynthesis [13]. A variety of *Dof* genes have been identified in plant genomes, with the number of *Dof* genes varying among different plants. For example, rice has 30 *Dof* family genes [14], *Arabidopsis thaliana* has 36 *Dof* family genes [14], soybean has 78 *Dof* family genes [15], and potato contains 35 *Dof* family genes [16]. Research has demonstrated that *Dof* gene family plays a crucial role in various biological processes related to plant growth and development, including plant growth regulation, signal transduction, seed germination, and abiotic stress [4]. In *Arabidopsis thaliana*, various conditions such as salt, drought, high temperature and abscisic acid induced the regulation of *AtDof3.3* gene expression, and there was a positive correlation between the high expression level of the *AtDof3.3* gene and the degree of tolerance to abiotic stress [17].The expression of *TaDof2, TaDof3*, and *TaDof6* genes significantly increased under drought and nitrogen stresses in wheat, suggesting a potential role of *TaDof* genes in response to abiotic stress [18]. *VcDof2* and *VcDof45* are believed to have significant roles in blueberry flowering and fruit development. Additionally, *VcDof1, VcDof11,* and *VcDof15* were found to respond positively and up-regulate their expression under abiotic stress, indicating potential roles in the defence of blueberries against such stress [19]. Tomato's *SlCDF3* is highly expressed under the influence of short sunlight, inducing the expression of *SlSP5G2* and *SlSP5G3*. The transcription of *SlSP5G* is induced under the influence of long sunlight, leading to late flowering in tomato [20]. The oil content of cotton seeds grown on land is linked to *GhDof1.* When a large amount of *GhDof1* is expressed, the oil content of the seeds increases while the protein content decreases [21]. The expression of *Dof* proteins in collard green rapeseed is affected by low temperatures. Under cold treatment, the expression of *BnCDF1* increases, and the constitutive overexpression of *BnCDF1* enhances the frost tolerance of plants [22]. Salt stress inhibits the expression of rice *OsDOF15* in the root system. On the other hand, overexpression of *OsDOF15* limits ethylene synthesis, which results in reduced root sensitivity to salt stress. This suggests that *OsDOF15-*mediated ethylene biosynthesis may be involved in the inhibition of primary root elongation by salt stress [23]. *IbDof2, IbDof16,* and *IbDof36* are up-regulated in response to abiotic stress sources and hormones, and may play a key role in stress tolerance [24]. The grape *VaDof17* gene overexpression has been found to enhance cold tolerance [25]. In chrysanthemum, *CmDOF16* is associated with root development, while *CmDOF20* and *CmDOF21* are significantly more expressed in reproductive organs than in nutrient organs, indicating their crucial role in reproductive development [26]. Additionally, CaDof6, CaDof14, CaDof16, and CaDof28 of chilli were found to be highly expressed in the root system [27]. The study presented above establishes the basis for identifying and analysing *Dof* gene family in *C.esculentus*at a genome-wide level.

Currently, research on cloning and functional studies of Dof proteins is primarily focused on common economic plants such as *Arabidopsis thaliana*, soybean, maize, rice, and wheat. However, there are few reports on related studies of *Dof* gene family in Cyperus species, particularly *C.esculentus*. One study found that *CEP2C19* enhances drought tolerance in *C.esculentus* by regulating ABA sensitivity [28]. Scholars combined PacBio High Fidelity (HiFi), Illumina Novaseq, and Hi-C technologies to perform the first high-quality and chromosome-scale genome assembly of *C.esculentus* [29]. The phylogeny of the *PP2C* gene family in *C.esculentus* was analyzed through a series of experimental manipulations, including RNA isolation and qRT-PCR, drought and ABA treatments of *C.esculentus* and *Arabidopsis* genes. The analyzes concluded that increased overexpression of *CePP2C19* affects drought tolerance, particularly in the early stage of seedlings, and significantly reduces ABA sensitivity [28]. Scholars have analyzed the physicochemical properties of *C.esculentus* seeds and found that they contain various substances, such as carbohydrates, starch, and saturated fatty acids [30]. In addition, transcriptomics and lipidomics were used by some scholars to track and analyze the key regulators of lipid biosynthesis during *C.esculentus* tuber development [31]. Scholars have studied the *CeDGAT2-2* protein of *C.esculentus* and found that it has high enzyme activity in catalysing TAG formation and strong specificity for oleic acid substrates. Overexpression of *CeDGAT2-2* in *C.esculentus* tubers enhances the accumulation of oil and oleic acid in the tobacco leaf [32].

*C.esculentus* is a promising cash crop, both as an important oil plant and as a concentrate feed for livestock. Current research on *C.esculentus* focuses on its nutrient composition, physicochemical properties, cultivation technology, and medicinal value [33–37]. Zhao et al.his indicates that *Dof* gene structure is conserved, which is consistent with the gene structure reported in lotsis [29]. However, no research has been conducted on the physicochemical properties, amino acid composition, secondary structure, or cis-acting elements of *Dof* family in *C.esculentus*. This study utilised bioinformatics methods to identify and analyze the members of the *Dof* gene family in the entire genome of *C.esculentus*. Additionally, we performed homology comparisons and constructed a phylogenetic tree with the members of *Dof* family in the genomes of ten other species, including *Arabidopsis thaliana*. We propose to explore the relationship and the evolutionary origins of *Dof* gene family in the genomes of between *C.esculentus* and other species. These results will provide a reference for future research on the functions of *Dof* gene family in *C.esculentus* and the breeding of *C.esculentus*.

## Materials and methods

### Data acquisition, experimental materials and design

The whole genome sequence, protein sequence, and gene annotation files of *C.esculentus* were downloaded from China National GeneBank DataBase (CNGBdb)(https://ftp.cngb.org/pub/CNSA/data5/CNP0003839/CNS0648185/CNA0051961/) [29] The genome sequences, protein sequences and annotation files of *Arabidopsis thaliana, Carex littledalei, Rhynchospora breviuscula, Rhynchospora pubera* and *Rhynchospora tenuis* were downloaded from National Genomics Data Center (https://ngdc.cncb.ac.cn/gwh/). The protein sequences and gene sequences of *Dof* gene family of *C.esculentus* and other species above were extracted from the protein sequences and genome sequences of their species using the TBtools tool after being identified as a *Dof* gene family by Pfam modelling of *Dof* gene families and SMART searches for Dof conserved domain, and all the *Dof* protein sequences and gene sequences were used for subsequent bioinformatics analysis.

*C. esculentus* seedlings are planted in Key Laboratory of Sichuan Province for Bamboo Pests Control and Resource Development of Leshan Normal University from July 1th to Nov 30th, 2023. Two experimental treatments were conducted on seedlings in laboratory pots, one involving drought stress and the other involving salt stress. The first experimental design is as follows:control groups(CK) were set up, and *Cyperus esculentus* seedlings were watered normally for 7 d, and the experimental group(T1,T2) were drought-treated with *O. latifolia* seedlings for 7 d and 14 d. The second experimental design is as follows: The group without NaCl added was designated as the control group (CK), while the groups with 3 g/L, 6 g/L, and 12 g/L NaCl added were designated as treatment groups (T1, T2, T3). The total RNA was extracted from young leaves of the treatment group and the control group. Reverse transcription of purified RNA into cDNA using a reverse transcription kit, and reverse transcribed cDNA was used for qRT-PCR to verify the expression of *Dof* transcription factor family members in *Cyperus esculentus*. Plant RNA extraction kit and cDNA reverse transcription kit are purchased from TIANGEN Biotech(Beijing)Co.,Ltd. The qRT-PCR experiments in this study were all completed on fluorescence quantitative PCR instrument (qToWer3 G) of the Analytick Jena AG. The number of replicates of biological samples in each treatment group and control group is 3, and the number of machine replicates on fluorescence quantitative PCR is 3. All *CesDof* gene primers designed by TBtools Batch q-RT-PCR primer design tool used in the qRT-PCR validation experiment in this study are shown in Supplementary Table 1. The qRT-PCR primers used in this study were synthesized by Sangon Biotech (Shanghai) Co., Ltd on commission.

### Identification, chromosomal localization and gene structure analysis of *CesDof* gene family

We analyzed conserved domains of CesDof proteins by using the online software SMART (http://smart.embl-heidelberg.de/) and identified family genes and simplified selected sequences by using Tbtools software. We carried out chromosomal localisation analysis of the *Dof* gene family by using MapChart software. The gene structure of *CesDof* genes was mapped and the exon and intron structure was analyzed using the GSDS 2.0 tool (http://gsds.cbi.pku.edu.cn/).

### Physicochemical properties analysis of CesDof proteins

The molecular weight, isoelectric point, instability index, total average hydrophobicity, and lipolysis coefficient of *CesDof* proteins were analyzed using the online tool Protparam (http://web.expasy.org/protparam/).

### Subcellular localization and signal peptide prediction of CesDof proteins

The subcellular localization and signal peptide of *CesDof* proteins were predicted by using the CELLO online software (http://cello.life.nctu.edu.tw/) and SignalP-5.0 Server (http://www.cbs.dtu.dk/services/SignalP/) online software, respectively.

### Transmembrane structure, hydrophilicity, and phosphorylation site prediction of CesDof proteins

The transmembrane structure of CesDof proteins was analyzed by using TMHMM Server v.2.0 software (http://www.cbs.dtu.dk/services/TMHMM/). Additionally, the hydrophobicity of CesDof proteins was predicted by using ProtScale (https://web.expasy.org/protscale/) and phosphorylation sites of CesDof proteins were analyzed using NetPhos3.1 Server (http://www.cbs.dtu.dk/services/TMHMM/).

### Secondary structure and conserved motif analysis of CesDof proteins

The CesDof proteins' secondary structure was predicted by using the SOPMA tool (https://npsa-prabi.ibcp.fr/cgi-bin/npsa_automat.pl?page=npsa_sopma.html). The MEME online tool (http://meme-suite.org/tools/meme) was used to analyze the *CesDof* proteins' conserved motifs, with the number of predictions set to 10 in the parameters and all other parameters set to default.

### Evolutionary tree analysis of CesDof proteins

Sequence comparison of CesDof protein sequences was performed using Clustalx. The phylogenetic tree of CesDof proteins was constructed using MEGA7 software with Neighbor-Joining (NJ) method and the calibration parameter BootStrap repeated 1000 times. The same method was used to construct the evolutionary tree of *C.esculentus* and *Arabidopsis thaliana*, as well as the evolutionary tree of *C.esculentus* and *Salvia officinalis*. Phylogenetic trees of *Dof* protein sequences from six species, including *C.esculentus*, *Arabidopsis thaliana, Rhynchospora tenuis, Rhynchospora pubera and Rhynchospora breviuscula*, were constructed using Maximum Likelihood (ML). The calibration parameters were repeated 1000 times, and bootstrap was repeated 1000 times.

### Analysis of cis-acting elements of *CesDof* genes

The promoter sequences of each member of the *Dof* gene family were selected from *C.esculentus* genome database. These sequences consist of 2000 bp upstream of the ATG. They were imported into text and collated into a fasta file. The file was then submitted to the PlantCARE website for promoter cis-acting element analysis. The sequences were filtered and sorted based on the results. Finally, they were visualised and analyzed using the 'Simple Bio Sequence Viewer' module in Tbtools.

### Transcription factor binding sites analysis of *CesDof* genes

The promoter sequences of each member of *Dof* gene family were obtained from *C.esculentus* genome database. A 2000 bp sequence upstream of the ATG start codon was used. The sequences were organized into a fasta file and submitted to PLANTREGMAP website for transcription factor binding site analysis. The data was sorted, filtered, and analyzed using the 'Simple Bio Sequence Viewer' in TBtools, following the analysis structure.

### Codon preference of *CesDof* genes

Relative synonymous codon usage (RSCU), codon adaptation index (CAI), GC content of synonymous codons (GC), Effective Number of Codon (ENC/Nc), Codon Bias Index (CBI), frequency of optical codons (Fop), and frequency of optimal codon usage (Fop) of CesDof genes were obtained by Codon W analysis.

### Collinearity and KaKs analysis of *CesDof* genes

Using default parameters, MCSCANX software was used to analyze the large segment tandem repeat events in *CesDof* gene family members. Based on the specific chromosome location information of the family members, TBtools software was used to analyze the collinearity of *Dof* genes in *C.esculentus* genome. The collinearity analysis between *C.esculentus* and *Arabidopsis thaliana, Carex littledalei, Rhynchospora tenuis, Rhynchospora pubera, a*nd *Rhynchospora breviuscula*. The simple Ka/Ks calculator of TBtools was used to analyze the KaKs of *CesDof* with collinearity.

### Analysis of SSR loci and microRNA target prediction for *CesDof* genes.

The SSR loci of *CesDof* genes were identified by using MISA-web (https://webblast.ipk-gatersleben.de/misa/) with modification parameters described by Gao et al. (2011). psRNATarget (https://www.zhaolab.org/psRNATarget/; Dai et al., 2018) with an expected value of 3 and default parameters, was predict to potential *CesDof* gene miRNAs from all miRNAs in the database.

### Protein–protein interactions analysis of CesDof proteins

The STRING interactive database was used to construct the interaction network between *CesDof* proteins. *Arabidopsis thaliana* was selected as the model plant to construct the protein interaction network diagram. The predicted network interactions were displayed by Cytoscape software.

Gene expression analysis of *CesDof* gene family.

The RNA extracted from all leaf samples in the two experimental groups and the control group under drought stress and salt stress conditions was reverse transcribed into cDNA and used for qRT-PCR verification experiments. The data obtained from the qRT-PCR experiments were statistically analyzed, and the gene expression data of *CesDof* gene family under drought stress and salt stress conditions were displayed as a histogram using the online analysis platform omicshare Tools (https://www.omicshare.com/tools/Home/Soft/histogram). The expression level changes of *CesDof* gene family under drought stress and salt stress conditions were analyzed.

## Results

### Gene structure and chromosomal localization of *Dof *gene family in *C.esculentus*

**Fig. 1:**
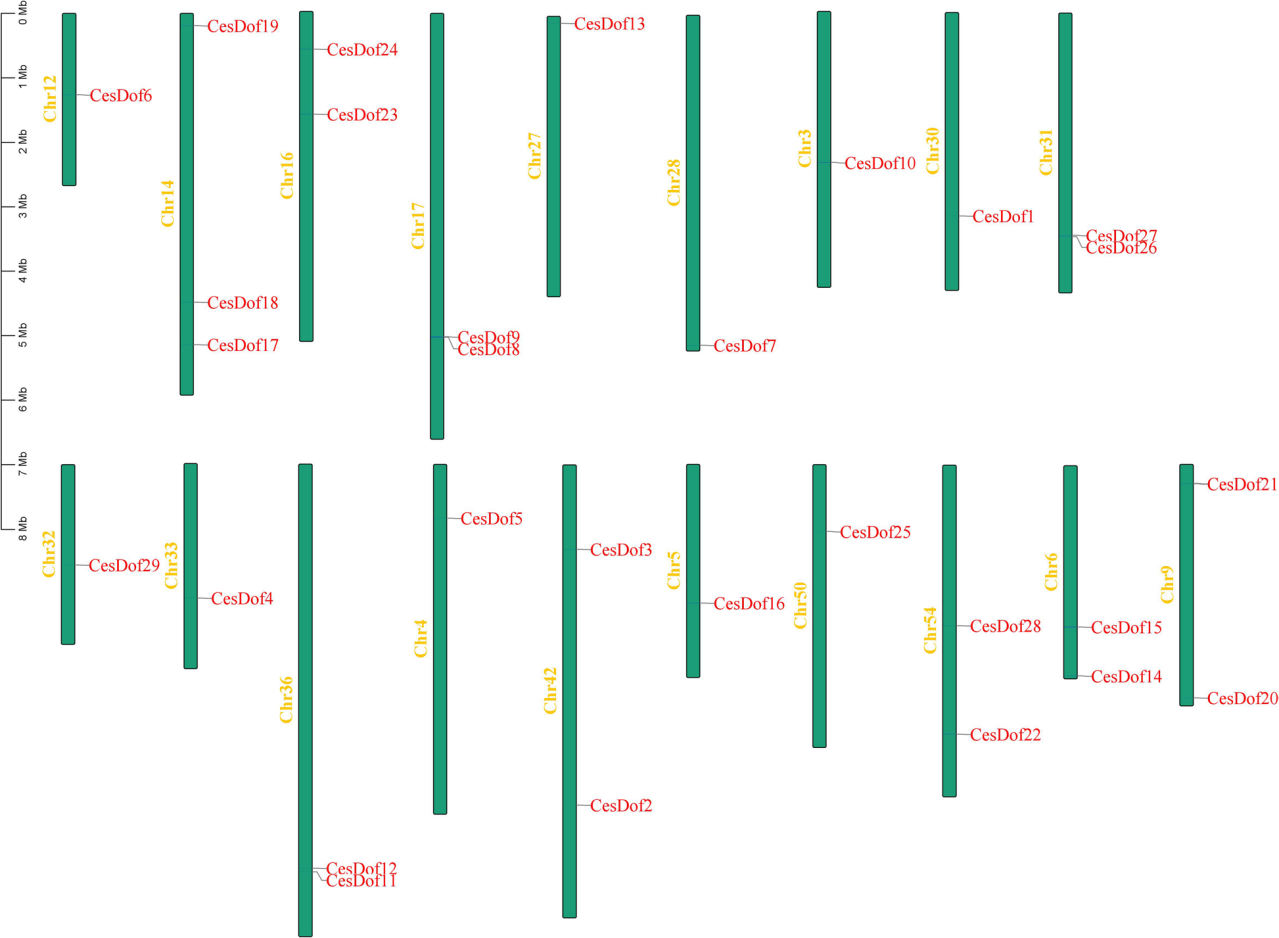
Chromosome mapping of* Dof *gene family in *C.esculentus*

The genome-wide identification results showed that there were 29 Ces*Dof* genes *C.esculentus* genome, which located on 19 chromosomes and named *CesDof01* ~ *CesDof29* according to the gene descriptions. Chr12, Chr27, Chr28, Chr3, and Chr30 contained *CesDof06, CesDof13, CesDof07, CesDof10,* and *CesDof1,* respectively. *CesDof17-CesDof19* were located on chromosome 14, while *CesDof23-CesDof24* were located on Chr16. *CesDof8-CesDof9* are located on chromosome 17. *CesDof29, CesDof4, CesDof5, CesDof16* and *CesDof25* are located on Chr32, Chr33, Chr4, Chr5, and Chr50, respectively. *CesDof11-CesDof12* are located on Chr36. *CesDof14-CesDof15* are located on chromosome 6. *CesDof20-CesDof21* are located on Chr9. *CesDof22* and *CesDof28* are located on Chr54. *CesDof26-CesDof27* are located on Chr31 (Fig. 1).

**Fig. 2:**
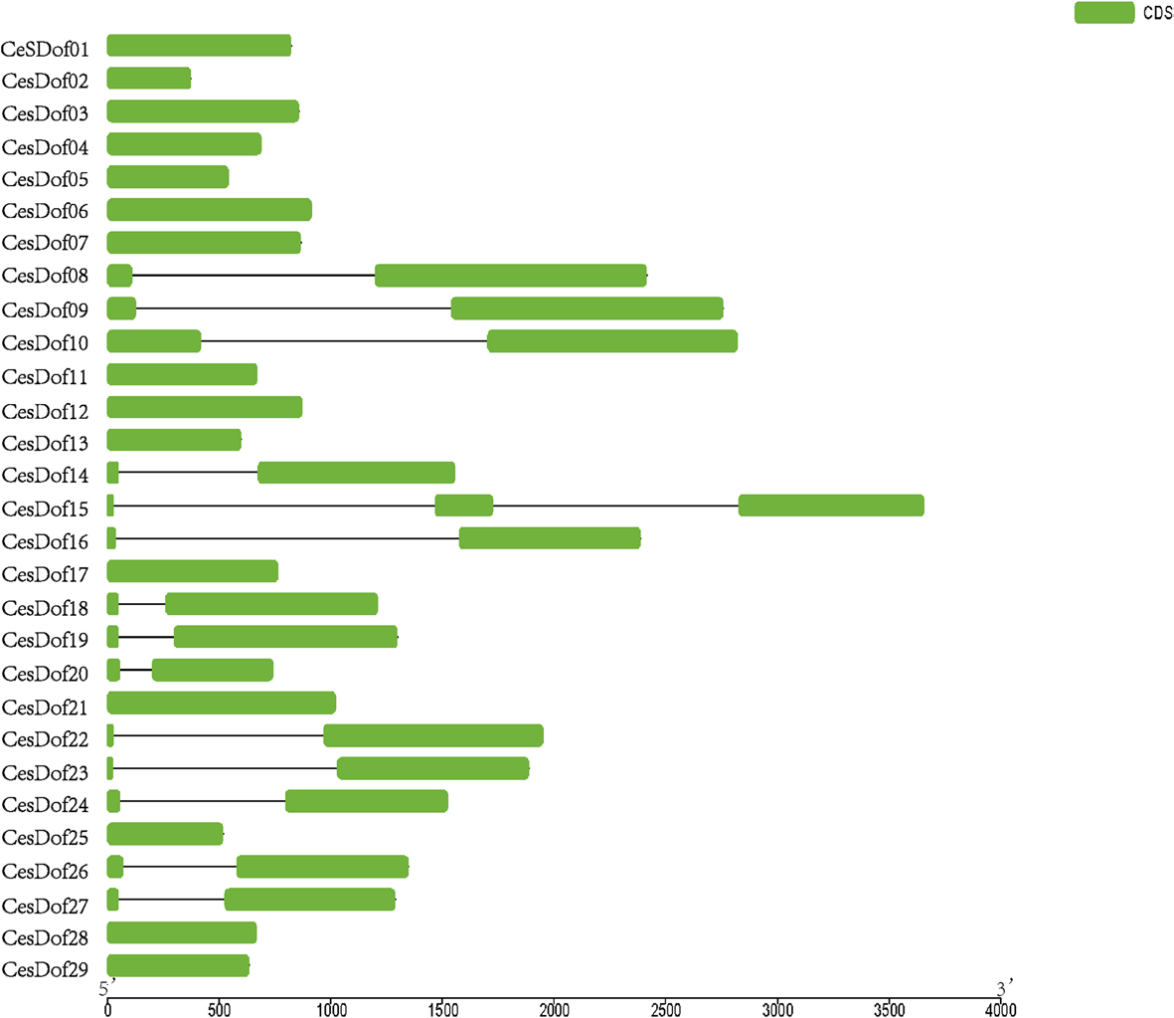
Gene structure of *Dof* genefamily in *C.esculentus*

The gene structure diagram of *CesDof* drawn by using the GSDS2.0 tool reveals that there are differences in sequence length and coding sequence length among the 29 *CesDof* gene sequences, with sequence lengths ranging from 400 to 3800 bp and coding sequence lengths ranging from 10 to 1000 bp. *CesDof08, CesDof09, CesDof10, CesDof14, CesDof16, CesDof18, CesDof19, CesDof20, CesDof22, CesDof23, CesDof24, CesDof26* and *CesDof27* have one intron and two exons, while the other *CesDof* gene sequences contain only one exon. *CesDof15* has 2 introns and 3 exons (Fig. 2).

### Physicochemical properties analysis of CesDof proteins

The physicochemical properties of CesDof proteins were analyzed. CesDof01 was found to have the highest number of amino acids, while CesDof25 had the lowest with 124 amino acids. The overall amino acid content of CesDof protein ranged from 124 to 512 amino acids, with an average of 286. The molecular weight of CesDof proteins ranged from 13.83 to 51.73 kD. The isoelectric point (pI) values ranged from 4.71 to 9.84. However, only 7 CesDof members had a theoretical isoelectric point less than 7, while the rest had a theoretical isoelectric point greater than 7. Therefore, most of CesDof members were basic proteins. Comparison of the instability index values showed that all CesDof sequences were predicted to be unstable proteins, except for CesDof09 and CesDof25 are stable proteins, which had instability index values below 40. All other CesDof sequences had instability index values greater than 40 and were predicted to be unstable proteins. The aliphatic index content of CesDof proteins ranged from 35.76 to 75.39, indicating a wide variation in thermal stability among this family of proteins. According to the prediction of protein hydrophilicity/hydrophobicity, all 29 CesDof members were found to be hydrophilic proteins, with negative GEAVY values. The highest hydrophilicity value (-0.344) was observed in CesDof11, while the lowest (-1.212) was observed in CesDof27*.* 6 CesDof proteins had a negative charge as the total number of negatively charged residues was greater than the total number of positively charged residues. The remaining 23 CesDof members had a positive charge as the total number of positively charged residues was greater than the total number of negatively charged residues (Table 1).

**Table 1:** Physicochemical properties of Dof proteins from *C.esculentus*

Protein	Amino acids number//aa	MW/kD	pI	instability index	Aliphatic index	Average of hydropathicity (GEAVY)	Asp + Glu	Arg + Lys	subcellular localization
CesDof01	512	51.73	9.36	51.98	60.96	-0.902	59	79	Nucleus
CesDof02	180	19.57	8.89	47.23	54.72	-0.526	12	17	Nucleus
CesDof03	282	30.59	8.07	46.66	65.43	-0.563	24	26	Nucleus
CesDof04	310	33.51	8.84	43.93	56.06	-0.593	18	23	Nucleus
CesDof05	371	38.96	8.10	46.19	56.58	-0.644	22	24	Nucleus
CesDof06	199	22.25	5.93	77.48	50.05	-0.699	20	18	Nucleus
CesDof07	340	35.29	7.63	48.60	55.21	-0.432	22	23	Nucleus
CesDof08	304	33.46	4.78	50.89	75.39	-0.399	33	24	Nucleus
CesDof09	254	28.06	8.96	34.28	59.49	-0.682	19	26	Nucleus
CesDof10	333	35.18	9.58	59.55	55.14	-0.543	17	27	Nucleus
CesDof11	350	36.37	9.46	55.11	57.00	-0.344	17	27	Nucleus
CesDof12	293	30.65	9.25	46.12	42.32	-0.670	15	22	Nucleus
CesDof13	261	28.51	9.53	40.96	70.23	-0.534	14	24	Nucleus
CesDof14	442	48.17	6.61	56.87	49.23	-0.770	48	46	Nucleus
CesDof15	448	48.70	8.20	45.95	58.15	-0.743	49	52	Nucleus
CesDof16	199	21.89	6.42	43.70	54.97	-0.764	20	18	Nucleus
CesDof17	288	30.96	8.89	45.91	64.62	-0.501	16	22	Nucleus
CesDof18	274	29.51	6.23	46.05	65.55	-0.445	26	23	Nucleus
CesDof19	280	29.57	9.34	46.19	55.79	-0.524	13	21	Nucleus
CesDof20	271	29.46	9.84	55.82	66.61	-0.563	13	23	Nucleus
CesDof21	211	23.02	4.71	45.35	43.51	-0.653	18	11	Nucleus
CesDof22	229	24.88	7.62	52.96	60.92	-0.571	20	21	Nucleus
CesDof23	223	24.18	8.74	60.58	67.31	-0.422	18	22	Nucleus
CesDof24	290	31.37	8.99	50.69	61.90	-0.437	13	20	Nucleus
CesDof25	124	13.83	7.61	37.77	50.32	-0.773	14	15	Nucleus
CesDof26	285	29.95	9.14	59.67	50.11	-0.549	19	24	Nucleus
CesDof27	172	19.05	9.14	70.43	35.76	-1.212	22	27	Nucleus
CesDof28	336	34.69	8.99	43.51	58.78	-0.362	21	26	Nucleus
CesDof29	222	24.56	6.32	55.06	54.14	-0.832	27	26	Nucleus

### Signal peptide prediction and subcellular localization results of CesDof proteins

All 29 *Dof* proteins of *C.esculentus* were predicted to lack signal peptides, indicating that they are non-secretory proteins. Additionally, the subcellular localization prediction showed that all 29 CesDof members were located to the nucleus. These results suggest that the *C.esculentus Dof* transcription factors primarily function in the nucleus (Table 1).

### Transmembrane structure, hydrophilicity and phosphorylation site prediction of CesDof proteins

The transmembrane structure prediction for the *CesDof* gene family in *C.esculentus* revealed that none of 29 CesDof proteins members exhibited transmembrane phenomena. Therefore, it was inferred that this family of proteins are non-transmembrane proteins.

The hydrophilicity/hydrophobicity analysis revealed that CesDof protein members had hydrophobicity values ranging from 0.833 to 2.767 and hydrophilicity values ranging from -4.056 to -2.311. The most hydrophobic member was CesDof01 with a value of 2.767, while the most hydrophilic member had a value of -4.056. The amino acid scores were inversely proportional to the hydrophilicity of the members, with lower scores indicating higher hydrophilicity and higher scores indicating higher hydrophobicity. CesDof01 has a hydrophobic alanine at position 112, and hydrophilic arginine and glutamic acid at positions 35, 36, and 37. Overall, CesDof proteins are hydrophilic, with more numerous and denser hydrophilic peaks than hydrophobic peaks (Table 2).

**Table 2:** Hydrophilicity / hydrophobicity analysis of *Dof* proteins in *C.esculentus*

Protein name	Maximum hydrophobicity			Maximum hydrophobicity		
	Position	amibo terminal	Value	Position	amibo terminal	Value
CesDof01	112	A	2.767	35, 36, 37	R E R	-4.056
CesDof02	143	G	1.811	77	K	-2.6
CesDof03	220	S	1.344	109	R	-3.33
CesDof04	5	S	1.467	250, 251	H N	-3.433
CesDof05	190	F	1.778	51, 52, 53, 54, 55, 56	Q Q N Q Q P	-3.289
CesDof06	132	E	1.500	25	E	-3.5
CeDof07	255	A	1.367	96	K	-2.889
CeDof08	228	L	1.556	109, 110	R A	-2.533
CesDof09	191	V	1.156	210, 211	E D	-3.078
CesDof10	200	F	1.722	143	R	-3.011
CesDof11	6	V	1.633	131	R	-3.022
CesDof12	172	S	1.356	48	R	-3.400
CesDof13	183	S	1.644	71	K	-2.589
CesDof14	263	A	1.433	77, 78, 79, 414	E H S G	-3.211
CesDof15	311	P	1.578	176	K	-2.922
CesDof16	129	D	1.678	89	K	-3.411
CesDof17	9	T	1.800	103	R	-3.600
CesDof18	28	V	1.678	107	R	-2.311
CesDof19	63	D	1.278	149	S	-2.833
CesDof20	43	P	1.500	136	K	-3.578
CesDof21	183	A	1.311	83	Q	-2.389
CesDof22	10	L	1.578	84	S	-2.656
CesDof23	119	S	2.178	27	E	-2.922
CesDof24	264	A	1.700	157	N	-2.478
CesDof25	14	F	2.400	24	Q	-3.144
CesDof26	113	A	1.767	6	R	-3.289
CesDof27	22, 23	F G	0.833	35	E	-2.678
CesDof28	263	A	1.589	54	P	-3.122
CesDof29	90–91	I E	1.311	8, 9	P R	-2.922

Phosphorylation site analysis of CesDof proteins revealed a total of 1141 serine (Ser) phosphorylation sites, 433 threonine (Thr) phosphorylation sites, and 147 tyrosine (Tyr) phosphorylation sites in the 29 CesDof members. Among CesDof protein members, CesDof01 has the highest number of serine (Ser) phosphorylation sites, with a total of 78. The most probable phosphorylation site is serine (Ser) at position 206, with a value of 0.998. CesDof01 also has the highest number of threonine (Thr) phosphorylation sites, with a total of 34. The most probable phosphorylation site is threonine (Thr) at position 420, with a value of 0. 875. CesDof21 has the highest number of tyrosine (Tyr) phosphorylation sites, with a total of 9. The most probable phosphorylation site is tyrosine (Thy) at position 193, with a value of 0.985. CesDof01 has the highest total number of phosphorylation sites, with a total of 78. *CesDof22* and *CesDof24* have the lowest total number of phosphorylation sites, with only two each. Given that the serine content is highest in this family, it is likely that the proteins primarily carry out their functions through phosphorylation at the serine (Ser) site (Table 3).

**Table 3:** Phosphorylation sites analysis of CesDof proteins

Protein name	Phosphorylation site			Serine (S)		Threonine (T)		Tyrosine (Y)	
	Serine (S)	Threonine (T)	Tyrosine (Y)	Max	Position	Max	Position	Max	Position
CesDof01	78	34	4	0.998	206	0.874	420	0.678	228
CesDof02	20	6	5	0.997	78, 79	0.904	74	0.894	106
CesDof03	30	8	8	0.991	28	0.901	84	0.987	155
CesDof04	35	23	6	0.998	37	0.968	22	0.925	9
CesDof05	57	16	6	0.996	130, 282	0.868	288	0.908	97
CesDof06	30	15	5	0.998	18	0.960	94	0.983	11
CesDof07	54	13	8	0.996	170	0.923	71	0.980	321
CesDof08	29	7	8	0.995	156	0.915	211	0.930	249
CesDof09	30	8	7	0.998	207	0.821	75	0.989	121
CesDof10	56	17	5	0.993	72	0.923	118	0.972	9
CesDof11	66	20	3	0.997	138	0.923	106	0.953	9
CesDof12	53	9	5	0.990	113	0.883	24	0.666	71
CesDof13	19	15	7	0.983	119	0.944	86	0.735	212
CesDof14	71	24	4	0.995	79	0.921	377	0.666	131
CesDof15	64	24	4	0.996	57, 59, 364	0.952	115	0.666	137
CesDof16	12	19	3	0.947	113	0.985	94	0.666	49
CesDof17	41	17	8	0.995	104	0.958	88	0.863	139
CesDof18	52	23	3	0.997	131	0.992	37	0.893	41
CesDof19	49	12	5	0.997	149	0.958	135	0.969	54
CesDof20	30	17	5	0.990	147	0.958	121	0.949	9
CesDof21	13	15	9	0.986	6	0.942	39	0.985	193
CesDof22	53	10	2	0.997	86, 89, 112	0.882	79	0.666	44
CesDof023	29	9	5	0.995	83	0.909	8	0.666	41
CesDof024	34	12	2	0.992	34	0.923	81	0.666	66
CesDof025	11	8	4	0.996	38	0.856	121	0.678	83
CesDof026	22	13	5	0.997	70, 74	0.846	193	0.666	32
CesDof027	27	14	3	0.997	72, 168	0.981	43	0.678	80
CesDof028	42	12	3	0.997	116	0.901	89	0.790	295
CesDof029	34	13	5	0.998	12	0.901	46	0.666	31

### Secondary structure of CesDof proteins

The prediction showed that irregular coil dominate in all 29 CesDof members, ranging from 57.47% (CesDof13) to 82.14% (CesDof28). This was followed by α-helix and extended chain, with proportions ranging from 6.55% (CesDof28) to 28.72% (CesDof24) and 6.21% (CesDof24) to 91.4% (CesDof11), respectively. β-turn accounted for the lowest proportion of 1.19% (CesDof28)-10.18% (CesDof26). Among them, CesDof02, CesDof04, CesDof05, CesDof08, CesDof09, CesDof11, CesDof16, CesDof17, CesDof18, CesDof20, CesDof21, CesDof28 and CesDof29 exhibited an random coils > extended chains > α-helix > β-turn, while the remaining 16 CesDof members displayed an random coil > α-helix > extended chain > β-turn (Supplementary Table 2 and Supplementary Fig. 1).

### Conserved structure of CesDof proteins

The conserved structure of CesDof proteins were analyzed using the online tool MEME, resulting in the identification of 10 independent conserved motifs. The lengths of these motifs ranged from 11 to 41 amino acids(Supplementary Fig. 2A). Of the 10 conserved motifs, motif1 is a single zinc finger structure (C2-C2) and is present in 29 family members. This suggests that it is a core component of the *Dof* proteins of *C.esculentus*. It is hypothesized that the different numbers and types of motifs in different *Dof* proteins may be due to their varying functions in the organism. Out of the 29 CesDof members, each one contains at least one conserved motif, with a maximum of eight. 20 CesDof members all contained motif1, while 2 CesDof members contained 9 other motifs, excluding motif2 (Supplementary Fig. 2B).

### Evolutionary tree analysis of CesDof proteins

**Fig. 3:**
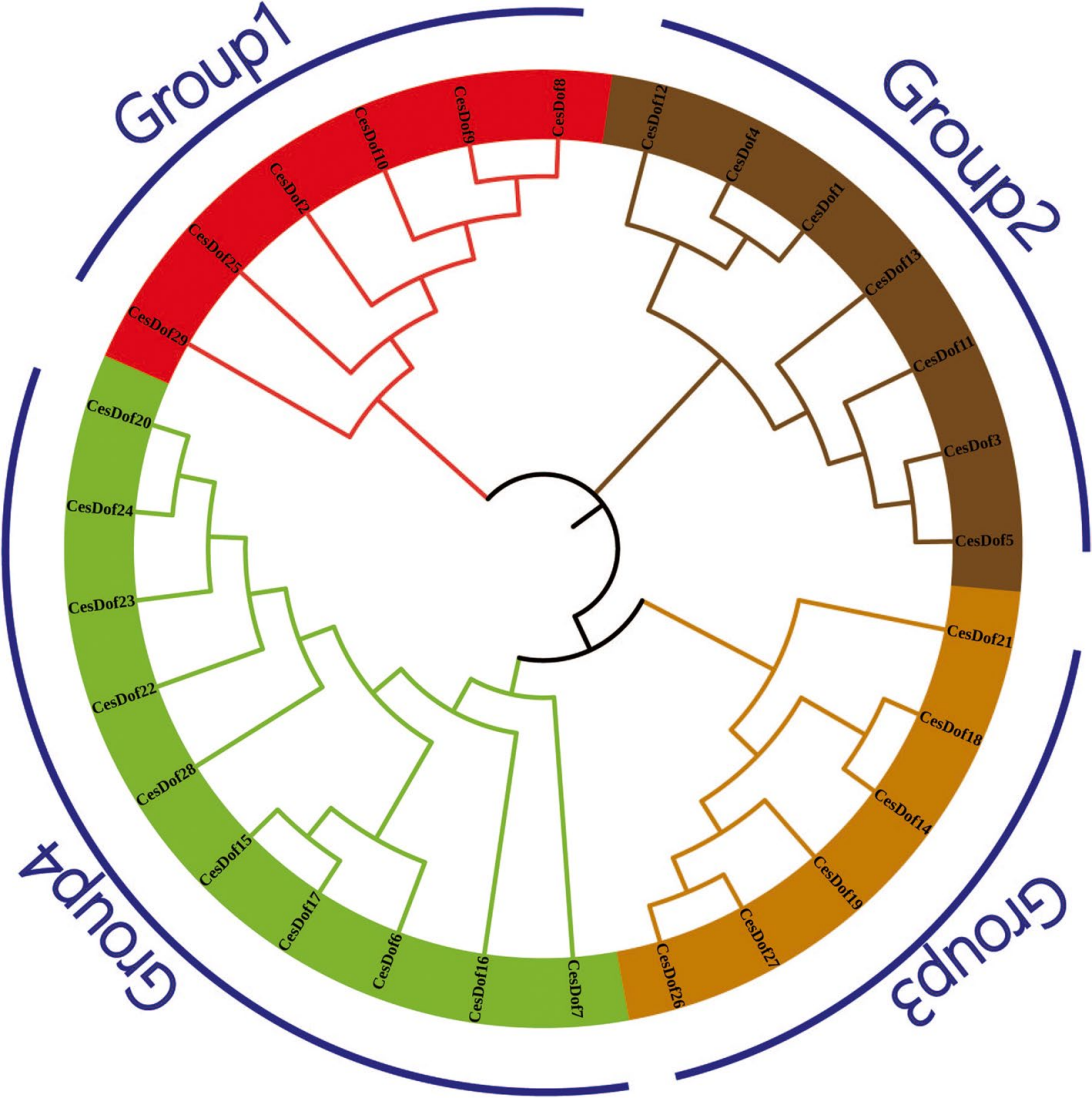
Phylogenetic tree of Dof proteins in *C.esculentus*

The 29 CesDof proteins can be divided into four distinct groups (labelled Group1, Group2, Group3 and Group4) based on their degree of aggregation in the tree. Group4 has the highest number of CesDof protein members, with 10 CesDof protein members, accounting for 34.48% of the total. Meanwhile, Group1 and Group3 have the lowest number of CesDof protein members, each with 6 CesDof protein members, accounting for 20.69% of the total. Group1 is considered the most primitive evolutionary group within the family, consisting of 6 CesDof protein members. Among them, CesDdof29 is the most primitive in terms of evolution, while CesDof08 is the fastest evolving sequence. Group4, on the other hand, is the fastest evolving group with 10 CesDof protein members. Among them, CesDof07 is the most primitive in terms of evolution, while CesDof20 is the fastest evolving (Fig. 3).

**Fig. 4:**
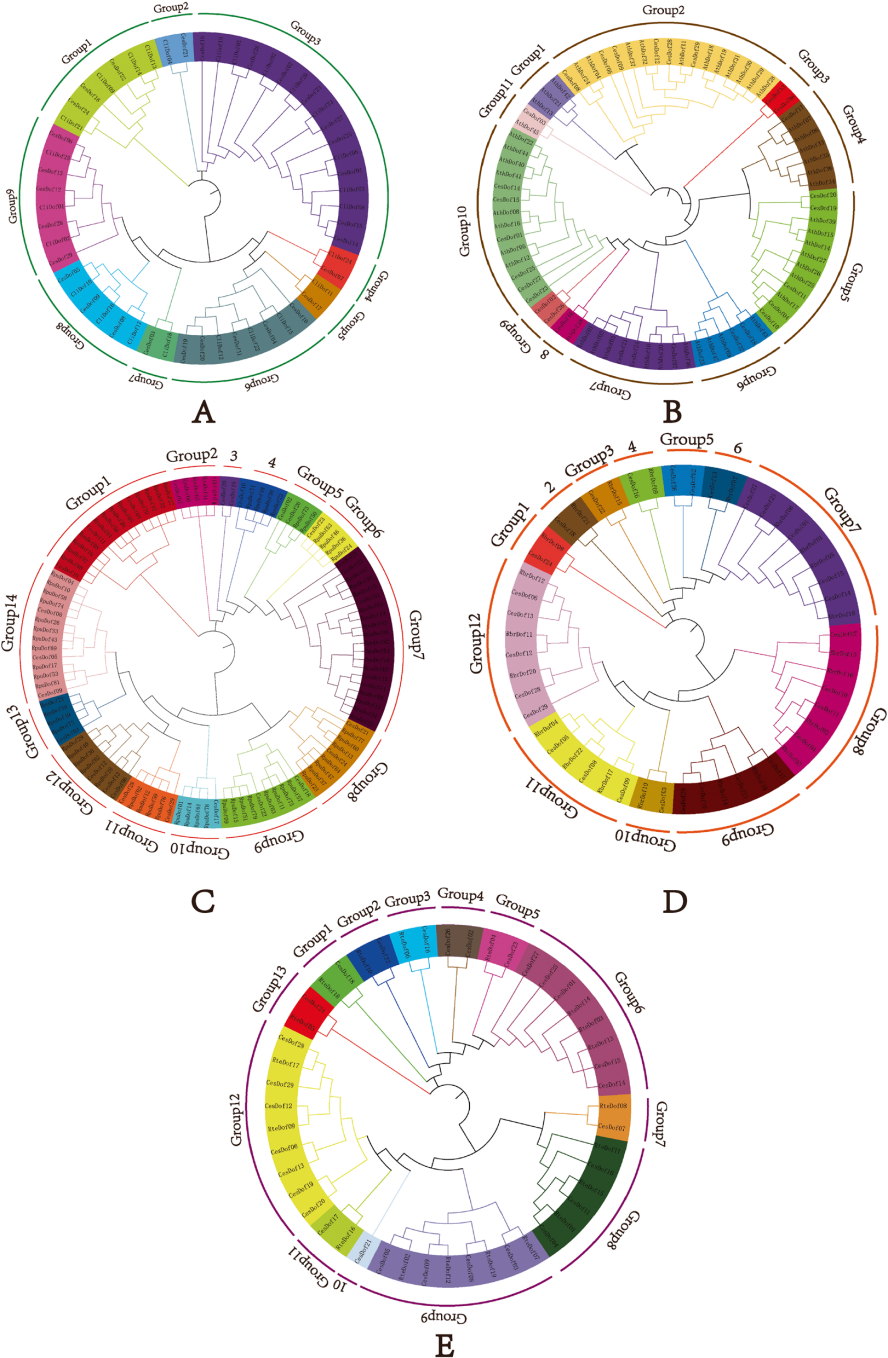
Molecular relationship analysis between *Dof *genes in *C.esculentus* and other species

The phylogenetic evolutionary tree of *Dof* gene family of *C.esculentus* and *Carex littledalei* reveals that the *Dof* proteins of both species can be classified into nine groups (Group1 to Group9). Group1 was the least evolved, consisting of 4 members of *C.esculentus* and 4 members of *Carex littledalei*. Among them, *CesDof24* and *CliDof21* were the most primitive, while *CliDof13* and *CliDof14* were the fastest to evolve. Group9, on the other hand, was the most evolved, comprising 5 members of *C.esculentus* and 3 members of *Carex littledalei*. *CesDof29* was the most primitive in Group9. Group 2 includes one member of *C.esculentus* and one member of *Carex littledalei*. Group 3 includes nine members of *C.esculentus* and eight members of *Carex littledalei*. Groups 4, 5, and 7 each contain one member of *C.esculentus* and one member of *Carex littledalei*. Group 6 includes one member of *C.esculentus* and one member of *Carex littledalei*. Group 7 also contains one member of *C.esculentus* and one member of *Carex littledalei*. Group 6 comprises five members of the *C.esculentus* family and three members of the *Carex littledalei* family. Group 8 consists of three members of the *C.esculentus* family and three members of the *Carex littledalei* family (Fig. 4A).

The phylogenetic evolutionary tree of *Dof* gene family in *C.esculentus* and *Arabidopsis thaliana* reveals that their phylogenetic relationships can be broadly classified into 11 groups (labelled sequentially as Group1, Group2, Group3, Group4, Group5, Group6, Group7, Group8, Group9, Group10, Group11). Group11 was the least evolved group, consisting of only one member from *C.esculentus* and one member from *Arabidopsis thaliana*. On the other hand, Group10 was the fastest evolving group, comprising of eight members from *Arabidopsis thaliana* and six members from *C.esculentus*. In Group 10, *CesDof23* is the most primitively evolved, while *AthDof22* and *AthDof44* are the fastest evolved. Group 1 contains three *Arabidopsis* members, Group 2 contains six *C.esculentus* members and eleven *Arabidopsis* members, Group 3 contains two *C.esculentus* members, and Group 4 contains six *Arabidopsis* members and one *C.esculentus* member. Group 5 comprises 7 members of *Arabidopsis* and 5 members of *C.esculentus*. Group 6 comprises 2 members of *C.esculentus* and 4 members of *Arabidopsis.* Group 7 comprises 6 members of *Arabidopsis* and 3 members of *C.esculentus*. Group 8 comprises 1 member of *C.esculentus* and 1 member of *Arabidopsis.* Group 9 comprises 2 members of *C.esculentus* exclusively (Fig. 4B).

According to the phylogenetic tree of *Dof* gene family in *C.esculentus* and *Rhynchopora pubera*, the phylogenetic relationship of *Dof* proteins in *C.esculentus* and *Rhynchopora pubera* can be roughly divided into 13 groups (labeled as Group 1, Group 2, Group 3, Group 4, Group 5, Group 6, Group 7, Group 8, Group 9, Group 10, Group 11, Group 12, Group 13). Overall, Group1 evolved the most primitive, including 12 *Rhynchopora pubera* members and 3 *C.esculentus* members; Group 14 has evolved the fastest, including 3 members of *C.esculentus* and 11 members of *Rhynchopora pubera*. Group 2, Group 6, Group 10, and Group 13 each contain 4 *Rhynchopora pubera* members and 1 *C.esculentus* member. Group3 contains 2 members of *C.esculentus*. Group4 contains 4 *Rhynchopora pubera* members and 1 *C.esculentus* member. Group 5 contains 2 members of *C.esculentus* and 2 members of *Rhynchopora pubera*, respectively. Group 7 contains 15 *Rhynchopora pubera* members and 5 *C.esculentus* members. Group8 contains 7 *Rhynchopora pubera* members and 2 *C.esculentus* members. Group9 contains 8 *Rhynchopora pubera* members and 2 *C.esculentus* members. Group 11 contains 4 *Rhynchopora pubera* members and 2 *C.esculentus* members. Group 12 contains 5 *Rhynchopora pubera* members and 3 *C.esculentus* members (Fig. 4C).

According to the phylogenetic tree of *Dof* gene family in *C.esculentus* and *Rhynchopora breviscula*, the phylogenetic relationship of *Dof* proteins in *C.esculentus* and *Rhynchopora breviscula* can be roughly divided into 12 groups (labeled as Group 1, Group 2, Group 3, Group 4, Group 5, Group 6, Group 7, Group 8, Group 9, Group 10, Group 11, Group 12 in sequence). Overall, the differentiation of Group 1 is relatively primitive, with one member from *C.esculentus* and one member from *Rhynchopora breviscula.* The Group12 group has the fastest evolution, with 5 members of *C.esculentus* and 3 members of *Rhynchopora breviscula*. Among them, *CesDof29* has the most primitive evolution, while *CesDof06* and *RbrDof12* have the fastest evolution. Group 2, Group 3, Group 4, Group 6, and Group 10 all contain one *C.esculentus* and one *Rhynchopora breviuscula* member. Group5 contains 2 members of *C.esculentus*. There are 5 members of *C.esculentus* and 3 members of *Rhynchopora breviscula* in Group 7. There are 4 members of *C.esculentus* and 4 members of *Rhynchopora breviscula* in Group 8. There are 4 members of *C.esculentus* and 2 members of *Rhynchopora breviscula* in Group 9. In Group 11, there are three members of *C.esculentus* and *Rhynchopora breviuscula*, respectively (Fig. 4D).

According to the phylogenetic tree of *Dof* gene family in *C.esculentus* and *Rhynchopora tenuis,* the phylogenetic relationship of *Dof* proteins in *C.esculentus* and *Rhynchopora tenuis* can be roughly divided into 13 groups (labeled as Group 1, Group 2, Group 3, Group 4, Group 5, Group 6, Group 7, Group 8, Group 9, Group 10, Group 11, Group 12, Group 13). Overall, Group12 evolved the most primitive, including two members, *CesDof24* and *RteDof05*. Group 13 has the fastest evolution, including 7 members of *C.esculentus* and 2 members of *Rhynchopora tenuis*. Among Group 13, *CesDof19* and *CesDof20* have the most primitive evolution, while *CesDof28* and *RteDof17* have the fastest evolution. Group 1, Group 2, Group 3, Group 5, Group 7, Group 11, and Group 13 each contain one *C.esculentus* member and one *Rhynchopora tenuis* member. Group 4 includes 2 members of *C.esculentus*. Group 6 includes 5 members of *C.esculentus* and 3 members of *Rhynchopora tenuis.* Group8 contains 3 members of *C.esculentus* and *Rhynchopora tenuis*, respectively. Group9 contains 4 members of *C.esculentus* and *Rhynchopora tenuis*, respectively. Group 10 only contains one *C.esculentus* member (Fig. 4E).

**Fig. 5:**
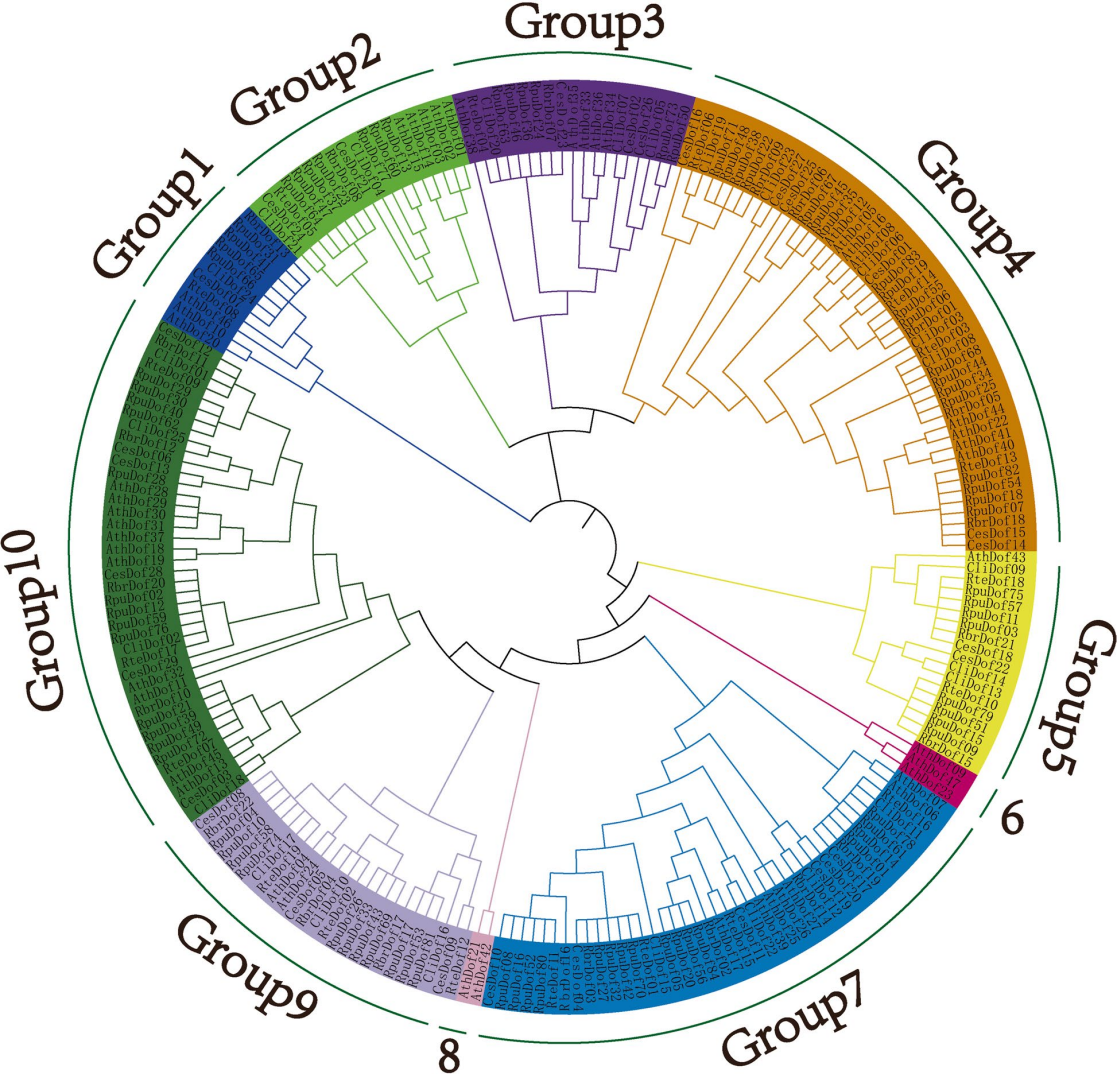
Phylogenetic tree of *Dof* gene family in* C.esculentus* andother species

According to the phylogenetic tree of *Dof* gene family between *C.esculentus* and five other species, the phylogenetic relationship of *Dof* proteins in *C.esculentus* and the other five species can be roughly divided into 10 groups (labeled as Group 1, Group 2, Group 3, Group 4, Group 5, Group 6, Group 7, Group 8, Group 9, Group 10). Overall, Group1 evolved the most primitive, consisting of three *Arabidopsis* members, one *C.esculentus* member, four *Rhynchopora pubera* members, one *Rhynchopora breviscula* member, and one *Rhynchopora tenuis* member. Group 10 has the fastest evolution, including 6 members of *C.esculentus*, 4 members of *Carex littledalei*, 11 members of *Arabidopsis*, 13 members of *Rhynchopora pubera,* 4 members of *Rhynchopora breviuscula*, and 3 members of *Rhynchopora tenuis*. Group 3 contains 3 members of *C.esculentus*, 5 members of *Arabidopsis*, 3 members of *Carex littledalei*, 1 member of *Rhynchopora tenuis*, 6 members of *Rhynchopora pubera*, and 1 member of *Rhynchopora breviscula*. Group 2 contains 2 members of *C.esculentus*, 5 members of *Arabidopsis,* 2 members of *Carex littledalei,* 7 members of *Rhynchopora pubera,* 1 member of *Rhynchopora tenuis*, and 1 member of *Rhynchopora breviscula*. Group 4 contains 6 members of *C.esculentus*, 8 members of *Arabidopsis,* 5 members of *Carex littledalei*, 2 members of *Rhynchopora tenuis*, 5 members of *Rhynchopora breviscula*, and 19 members of *Rhynchopora pubera*. Group 5 contains 2 members of *C.esculentus*, 1 member of *Arabidopsis*, 3 members of *Carex littledalei*, 2 members of *Rhynchopora tenuis*, 2 members of *Rhynchopora breviscula*, and 8 members of *Rhynchopora pubera*. Group6 only contains 3 *Arabidopsis* members. Group 7 contains 6 members of *C.esculentus*, 7 members of *Arabidopsis*, 4 members of *Carex littledalei*, 4 members of *Rhynchopora tenuis,* 5 members of *Rhynchopora breviscula*, and 16 members of *Rhynchopora pubera*. Group8 contains 2 *Arabidopsis* members. Group 9 contains 3 members of *C.esculentus*, 2 members of *Arabidopsis*, 3 members of *Carex littledalei,* 3 members of *Rhynchopora tenuis*, 3 members of *Rhynchopora breviscula*, and 11 members of *Rhynchopora pubera* (Fig. 5).

### Analysis of cis-acting elements of *CesDof* genes

**Fig. 6:**
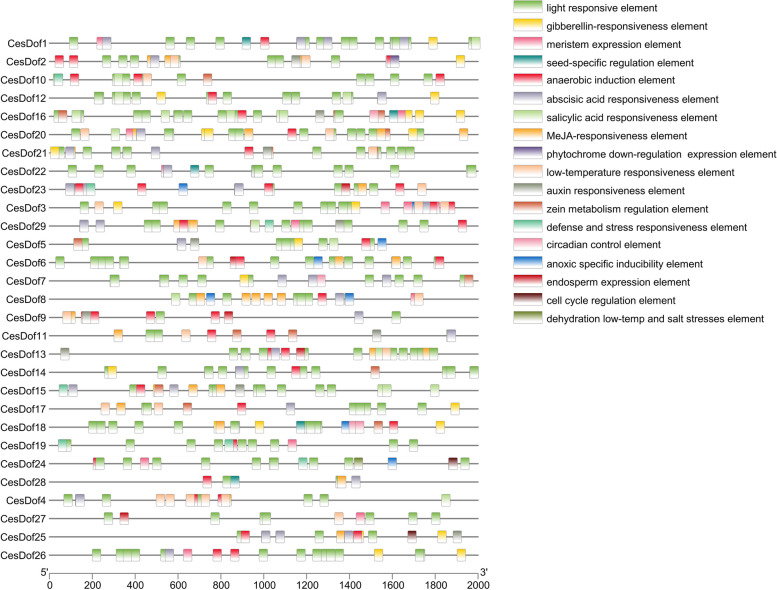
Cis-elements in promoters of *Dof *gene family in *C.esculentus*

The cis-acting elements analysis results of *CesDof* genes showed that besides the common *TAT-box* and *CAAT-box*, *C.esculentus* also contains cis acting elements that respond to light, drought, hormones, low temperature, and circadian rhythms. All member promoters contain a light responsive element. *CesDof02, CesDof03, CesDof05, CesDof07, CesDof12, CesDof14, CesDof15, CesDof16, CesDof17, CesDof18, CesDof20, CesDof21, CesDof25* and *CesDof26* contain a gibberellin responsive element. *CesDof01, CesDof02, CesDof03, CesDof26, CesDof08, CesDof16, CesDof19, CesDof20, CesDof22, CesDof24, CesDof26, CesDof27* and *CesDof29* contain meristem expression elements. *CesDof01, CesDof16, CesDof18, CesDof22, CesDof23* and *CesDof28* contain seed specific regulatory elements. Only *CesDof24* contains dehydration low temperature and salt stress elements. *CesDof10, CesDof15, CesDof19, CesDof28 Dof24, CesDof23* and *CesDof29* contain defense and stress response elements. *CesDof03, CesDof05, CesDof06, CesDof08, CesDof18, CesDof23* and *CesDof24* contain anoxic specific index elements. *CesDof02, CesDof05, CesDof11, CesDof15, CesDof21, CesDof09, CesDof13, CesDof16, CesDof29* and *CesDof25* contain auxin response element. *CesDof24* and *CesDof25* contain cell cycle regulation element. *CesDof07, CesDof16* and *CesDof18* contain circadian control element. Only *CesDod02* contains phytochrome down regulation expression element. *CesDof06, CesDof09, CesDof13, CesDof23* and *CesDof27* contain endosperm expression element. *CesDof01, CesDof16, CesDof18, CesDof22, CesDof23* and *CesDof28* contain seed specific regulation element, and other response elements are also widely present in *Dof* family genes of *C.esculentus* (Fig. 6).

### Transcription factor binding sites of *CesDof* genes

**Fig. 7:**
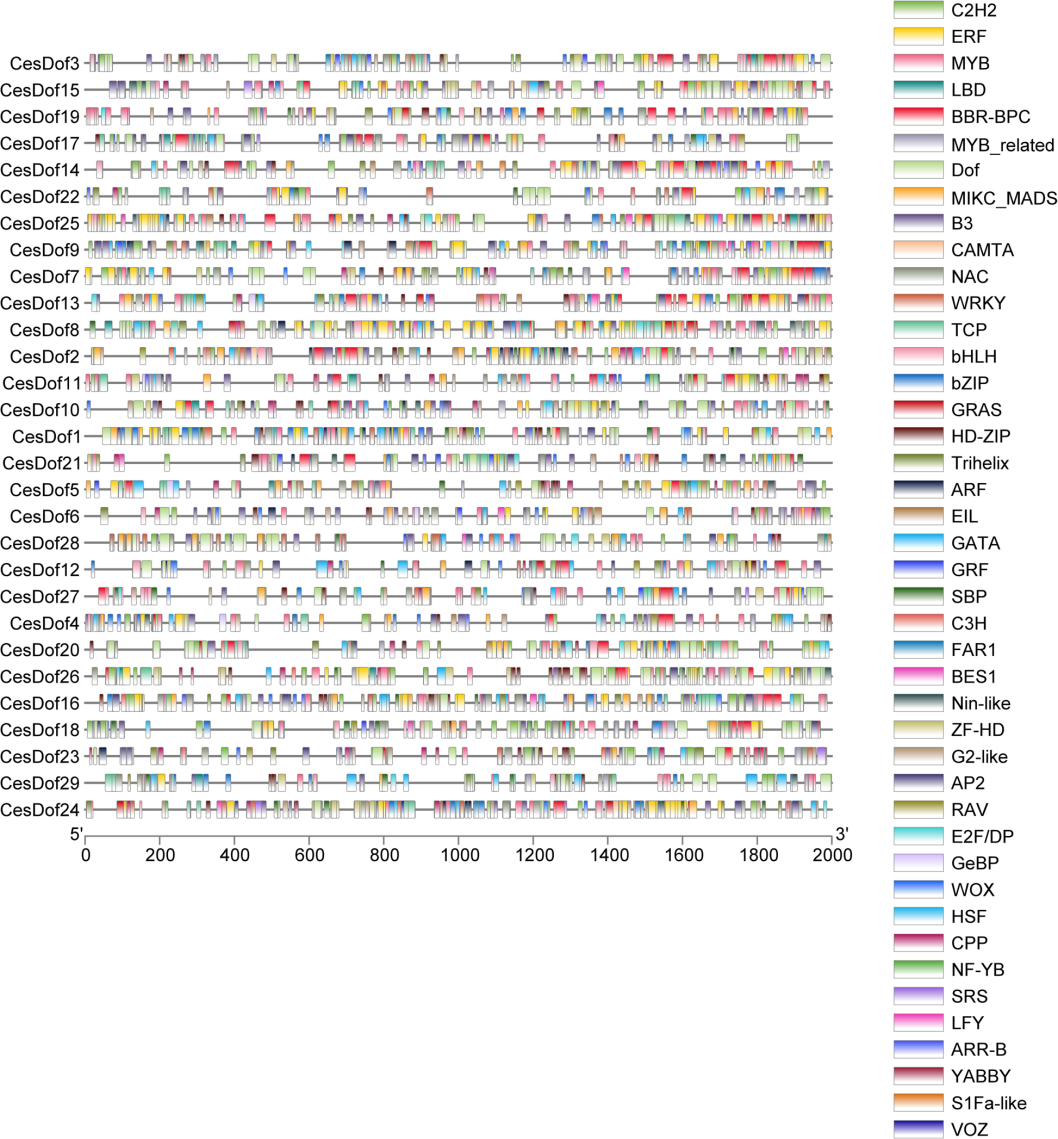
Transcription factor binding site of* Dof* gene family in *C.esculentus*

Tbtools was used to predict the transcription factor binding sites of *CesDof* gene family. The results showed that there were *C2H2, ERF, MYB, LBD, BBR-BPC, MYB_related, Dof, B3, CAMTA, NAC, TCP, WRKY, WOX, LFY, VOZ, ARR-B* transcription factor binding sites in *CesDof* genes. *Dof, ERF, MYB, BBR-BPC, MIKC_MADS* in *CesDof* genes was the most binding sites for these five transcription factors. Among them, in *CesDof* transcription factors are involved in various biological processes during plant growth and development, regulating light response and carbon and nitrogen metabolism, seed development and germination, plant hormone response, photosensitive pigment response, and defense response. *ERF* transcription factors not only act as ethylene responsive elements, but also exist in many genes that respond to low temperature or drought induction. *MYB* transcription factors are a class of transcription factors with *MYB* domains that can bind to the promoter region upstream of *DNA,* regulate gene transcription, and play important roles in flower growth and development, secondary metabolism, stress response, and other aspects. The *BBR-BPC* transcription factor can regulate the expression of inducible genes, producing various cis acting elements such as stress, plant hormones, tissue-specific expression, and photoresponse. *MIKC_ MADS* box family genes are very common in plants and can regulate plant growth and development, such as flower development and fruit ripening (Fig. 7).

### Codon preference analysis of *CesDof* genes

The codon preference analysis of different codons for each amino acid (RSCU), codon adaptation index (CAI), synonymous codon GC content (GC), and 3rd base content (GC3s, T3s, C3s, A3s, G3s), as well as the effective number of codons (ENC/Nc) and codon bias index (CBI) were obtained using CondonW analysis. The results show that CAI ranges from 0 to 1, with lower codon preference degrees resulting in CAI values closer to 0. The codon preference ranges from a minimum of 0.135 for *CesDof11* to a maximum of 0.275 for *CesDof29*. The Nc value ranges from 41.31 *(CesDof25)* to 57.81 *(CesDof26)*, with larger values indicating weaker codon preference and smaller values indicating stronger codon preference. The GC content, which is the total amount of GC in the codon, ranged from 0.671 (*CesDof15*) to 0.362 (*CesDof25*), with an average content of 0.495. The GC3s content ranged from 0.763 (*CesDof15*) to 0.331 (*CesDof25*), with an average content of 0.496. The codon 3rd base contents were T (0.329) > C (0.326) > G (0.287) > A (0.286). The content of T/A was higher than the content of G/C, indicating that the *CesDof* gene preferred to end in T/A. The CBI index was used to assess the potential expression of exogenous genes in the target hosts. A value of 1 indicates that all codons are optimally used, while a value of 0 indicates that all codons are used randomly. The range of CBI is from -0.117 (*CesDof25*) to 0.304 *(CesDof26).* The Fop value ranges from 0 to 1, with 1 indicating the use of only optimal codons and 0 indicating the absence of optimal codons. The content ranges from 0.332 (*CesDof25*) to 0.598 (*CesDof25*)(Supplementary Table 3).

The RSCU values of the codons in the *CesDof* gene family of *C.esculentus* analyzed using CodonW software. The results showed that 30 codons had RSCU values greater than 1, with only 7 of them ending in G/C. AGA was the most strongly preferred codon for *C.esculentus*, while AUG, AUA, GGC, and UGG had no codon preference (RSCU = 1). Additionally, 30 codons, including CUC, CUA, and CUG, had very low codon preference (RSCU < 1) (Supplementary Table 4).

### Colinearity analysis of *CesDof* genes

**Fig. 8:**
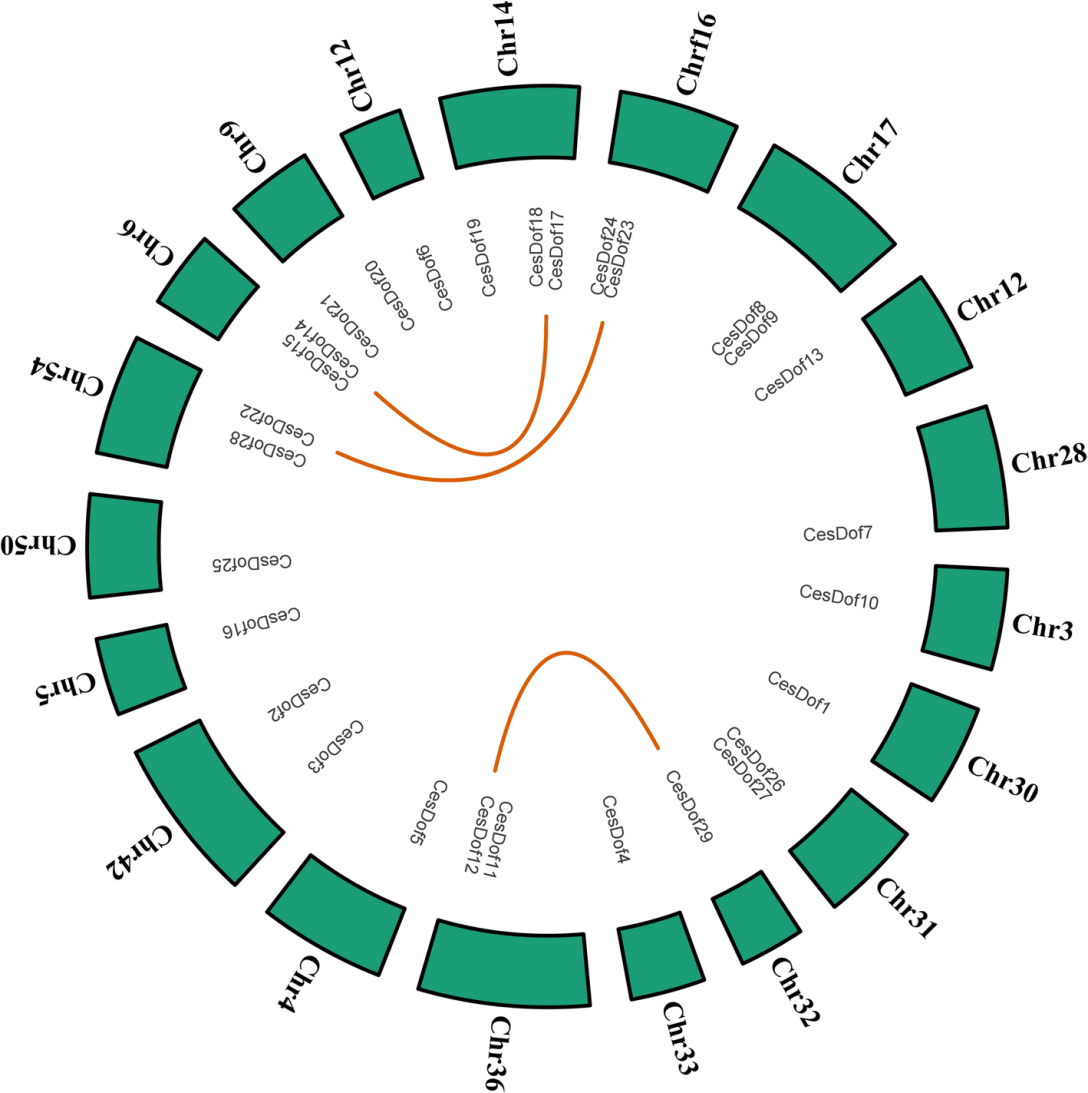
Colinearity analysis of *CesDof* genes in *C.esculentus*

TBtools was used to map the collinearity map of *CesDof* genes. The results showed that 29 *CesDof* genes constituted three pairs of collinearity genes, namely *CesDof22* and *CesDof*23, *CesDof*14 and *CesDof*17, *CesDof*29 and *CesDof*12. *CesDof*22, *CesDof*23, *CesDof*14, *CesDof*17, *CesDof*29, and *CesDof*12 are distributed on chromosomes Chr54, Chr16, Chr6, Chr14, Chr32, and Chr36, respectively (Fig. 8).

The ratio of nonsynonymous substitutions (Ka) to synonymous substitutions (Ks) can indicate whether selection pressure acted on the protein-coding gene in the plant. The Ka/Ks ratios for the *CesDof* replication events ranged from 0.21 (*CesDof22/CesDof29*) to 0.35 (*CesDof24/CesDof12*), all less than 1, suggesting that all *CesDof* replication events underwent purifying selection. *CesDof* is evolutionarily conserved (Table 4).

**Table 4:** The Ka/Ks ratios of *CesDof* duplication events in *C.esculentus*

Seq_1	Seq_2	Ka	Ks	Ka_Ks
CesDof22	CesDof17	0.560672109	NaN	NaN
CesDof23	CesDof29	0.616429403	2.956671636	0.20848761
CesDof24	CesDof12	0.673318744	1.947194096	0.345789228

**Fig. 9:**
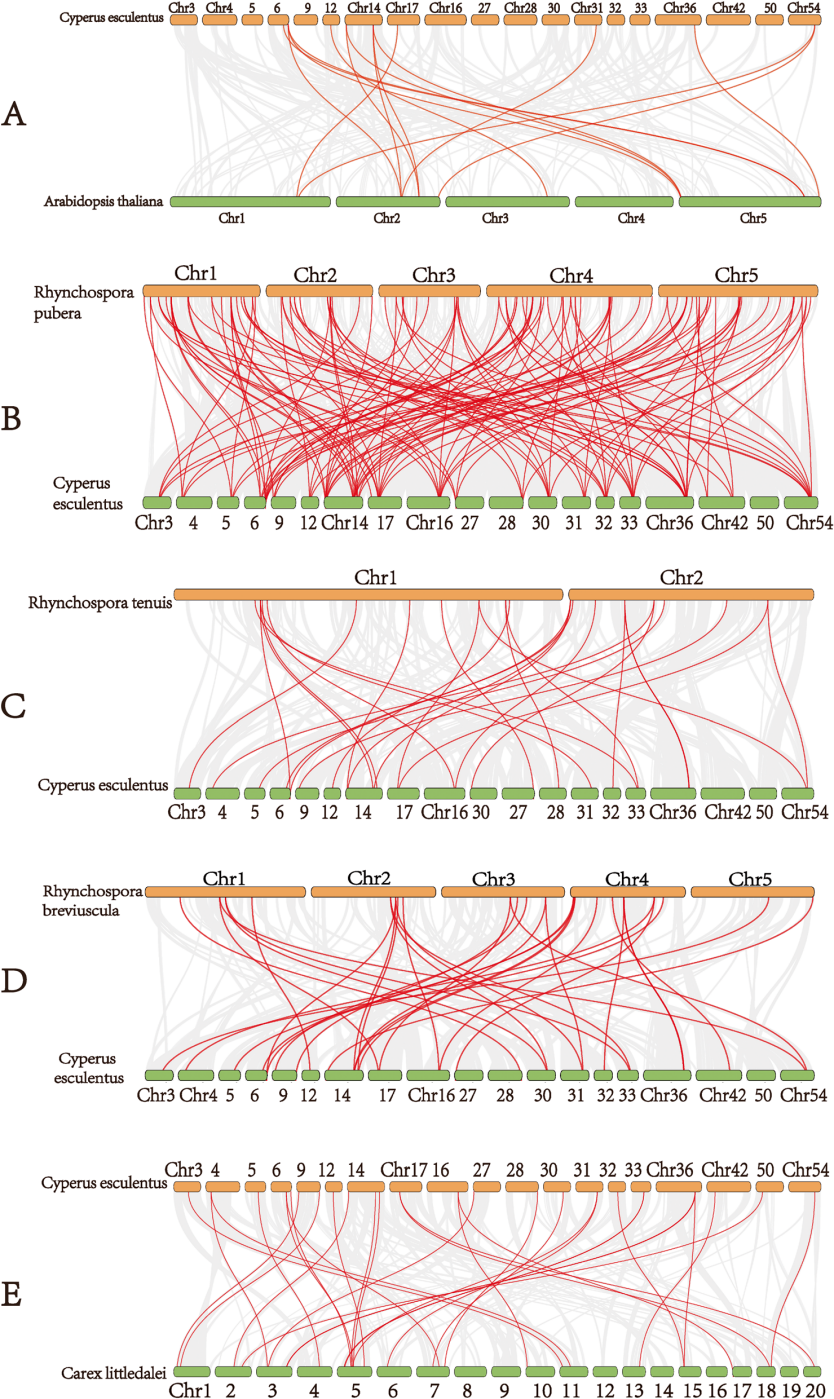
Colinearity analysis between *Dof *genes in *C.esculentus* and other species

Figure. 9A shows the five chromosomes of *Arabidopsis,* and the chromosomes of *C.esculentus* are represented by Chr3, Chr5, Chr9, Chr12, Chr14, Chr16, Chr17, Chr27, Chr28, Chr30, Chr33, Chr36, Chr42, Chr50, and Chr54. The grey lines indicate all the co-lined gene pairs of *Arabidopsis* and *C.esculentus*, while the red lines represent *Dof* genes. The results indicate that *Dof *members from *Arabidopsis* and *C.esculentus* formed a total of 15 colinear gene pairs. *C.esculentus* has 4, 1, 4, 1, 1, 1 and 2 collinearity gene pairs on chromosomes Chr6, Chr12, Chr14, Chr16, Chr31, Chr36, and Chr54, respectively. *Arabidopsis thaliana* has 2, 6, 1, 1, and 5 collinearity gene pairs on chromosomes Chr1, Chr2, Chr3, and Chr5, respectively. *C.esculentus* has the most collinearity gene pairs on chromosomes Chr6 and Chr14, both with 4 pairs, and the least collinearity genes on chromosomes Chr12, Chr17, Chr31, and Chr36, all with only 1 pair. *Arabidopsis thaliana *has the most collinearity gene pairs on chromosome Chr2, with six pairs, and the least collinearity genes on Chr3, with only one pair. *CesDof14* and *CesDof18* have the most collinearity gene pairs. *CesDof14*, located on Chr6, is collinearityly related to all of the *Arabidopsis* genes rna58242, rna46874, and rna21024, respectively. Similarly, *CesDof18*, located on Chr14, has collinearity with Arabidopsis genes rna46874, rna22952, and rna21024, respectively. *CesDof19* and* CesDof22*, located on chromosomes 14 and 54 respectively, have two collinearitys genes in *Arabidopsis* genes. These genes belong to a one-to-many collinearity relationship. On the other hand, *CesDof*06, *CesDof11,* *CesDof15*, *CesDof23* and *CesDof27* have only one homologous gene each in *Arabidopsis thaliana*, namely rna34843, rna59759, rna58242, rna12054, and rna21024, respectively. These genes belong to a one-to-one collinearity relationship (Fig.9A).

Figure 9B shows the five chromosomes of *Rhynchospora pubera,* represented by Chr1-Chr5. Additionally, Chr3-Chr5, Chr9, Chr12, Chr14, Chr16, Chr17, Chr27, Chr28, Chr30-Chr33, Chr36, Chr42, and Chr50 are in *C.esculentus*. The chromosomes of *C.esculentus* are represented by Chr54. The grey lines indicate co-linear gene pairs between *Rhynchospora pubera* and *C.esculentus*, while the red lines indicate *Dof* genes. The study found that *Dof* members from *Rhynchospora pubera* and *C.esculentus* comprised a total of 129 colinear gene pairs. All chromosomes in *Rhynchospora pubera* had colinear gene pairs, while in *C.esculentus*, all chromosomes had co-linear gene pairs except for chromosome Chr50. *Rhynchospora pubera*'s Chr1 had the highest number of co-linear gene pairs with 34, while Chromosome Chr5 had the lowest number with only 15 pairs. In *C.esculentus*, Chromosome Chr14 had the highest number of co-linear gene pairs with 22, while chromosomes Chr3, Chr4, Chr5, Chr12, Chr27, Chr28, and Chr31 had the lowest number with only 4 pairs each. The genes of *Rhynchospora pubera* and *C.esculentus* showed a high degree of homology. *CesDof12*, located on Chr36, had the highest number of homologous genes with 11, followed by *CesDof19* on Chr14 with 9 *Rhynchospora pubera* homologous genes. There are 8 *Rhynchospora pubera* homologous genes located in *CesDof04* on Chr33, *CesDof09* on Chr17, *CesDof14* on Chr6, *CesDof18* on Chr14, and *CesDof23* on Chr16, respectively. *CesDof05* located at Chr4, *CesDof06* located at Chr12, *CesDof07* located at Chr28, *CesDof10* located at Chr3, *CesDof13* located at Chr27, *CesDof15* located at Chr6, *CesDof16* located at Chr5, and *CesDof27* located at Chr31 have four *Rhynchospora pubera* homologues, respectively. *CesDof17* at Chr14 has five *Rhynchospora pubera* homologous genes. *CesDof01* located at Chr30 and *CesDof21* located at Chr9 have 6 *Rhynchospora pubera* homologs, respectively. *CesDof22* located at Chr54 and *CesDof29* located at Chr32 have 7 *Rhynchospora pubera* homologous genes, respectively. *CesDof02* at Chr42 has 3 *Rhynchospora pubera* homologous genes. *CesDof03* at Chr42 has 2 *Rhynchospora pubera* homologous genes. The above genes belong to one-to-many collinearity. *CesDof24* located in Chr16 has 1 *Rhynchospora pubera* and is r1.3G01104230.1, which is a one-to-one collinearity (Fig. 9B).

Figure 9C shows the chromosomes of *Rhynchospora tenuis* and *C.esculentus*, with grey lines indicating co-localised gene pairs and a red line indicating the *Dof* gene. The chromosomes are labelled as follows: Chr1, Chr2 for *Rhynchospora tenuis* and Chr3-Chr5, Chr9, Chr12, Chr14, Chr16, Chr17, Chr27, Chr28, Chr30-Chr33, Chr36, Chr42, Chr50, and Chr54 for *C.esculentus*. The study found that *Dof* members from *Rhynchospora tenuis* and *C.esculentus* formed a total of 28 collinearity gene pairs, these pairs were located on chromosome Chr11 and Chr2 in Rte. With co-linear genes present on all chromosomes in *C.esculentus* except for Chr42 and Chr50. On *C.esculentus*, there were up to five pairs of collinearity genes on chromosome Chr14, while only one pair was found on Chr12, Chr27, Chr28, Chr3, Chr30, Chr31, Chr32, Chr4, Chr5, and Chr9. The genes *CesDof04, CesDof09, CesDof14, CesDof18, CesDof19, CesDof22* and *CesDof23* of the *C.esculentus* plant are located on chromosomes 33, 17, 6, 14, 14, 54, and 16, respectively, each of these genes contains 2 collinearity genes of *Rhynchospora tenuis*. *CesDof01* located on Chr30, *CesDof05* located on Chr4, *CesDof06* located on Chr12, *CesDof07* located on Chr28, *CesDof10* located on Chr3, CesDof36 located on Chr36, CesDof11, CesDof12 located on Chr36, CesDof13 located on Chr27, *CesDof15* located on Chr6, *CesDof16* located on Chr5, *CesDof17* located on Chr14, *CesDof21* located on Chr9, *CesDof27* located on Chr31 and *CesDof29* located on Chr32 all contain only one *Rhynchospora tenuis* collinearity gene. The above genes belong to one-to-one collinearity (Fig. 9C).

Figure 9D shows the five chromosomes of *Rhynchospora breviuscula* (Chr1-Chr5) and the chromosomes of *C.esculentus* (Chr3-Chr5, Chr9, Chr12, Chr14, Chr16, Chr17, Chr27, Chr28, Chr30-Chr33, Chr36, Chr42, Chr50, and Chr54). The grey lines represent all co-lined gene pairs of *Rhynchospora breviuscula* and *C.esculentus*, while the red lines represent *Dof* genes.The study found that *Dof* members from *Rhynchospora breviuscula* and *C.esculentus* formed a total of 33 collinearity gene pairs. Collinearity genes were present on every chromosome of *Rhynchospora breviuscula*, with the highest number of collinearity pairs on CHr4 (12 pairs), and the lowest number of collinearity pairs on Chr5 (only 1 pair). In *C.esculentus*, all chromosomes except chromosome Chr50 had collinearity genes. Chromosome CHr14 had the most with 6 collinearity gene pairs, while Chr4, Chr5, Chr32, Chr3, Chr28, Chr27 and Chr12 had the least with only 1 collinearity gene pair each. The genes *CesDof01* at Chr30, *CesDof04* at Chr33, *CesDof09* at Chr17, *CesDof14* at Chr6, *CesDof17* at Chr14, *CesDof27* at Chr31, *CesDof23* at Chr16 and *CesDof22* at Chr54 have 2 collinearity *Rhynchospora breviuscula* genes. There are three co-linear genes between *CesDof18*, located in Chr14, and *Rhynchospora breviuscula*. All of the *C.esculentus* mentioned above are related to the collinearity relationship with one to many. The genes *CesDof2* at Chr42, *CesDof05* at Chr4, *CesDof06* at Chr12, *CesDof07* at Chr28, *CesDof10* at Chr3, *CesDof11* and *CesDof12* at Chr36, *CesDof13* at Chr27, *CesDof6* at Chr6, *CesDof15* at Chr5, *CesDof16* at Chr5, *CesDof19* at Chr14, *CesDof20* and *CesDof21* at Chr9, and *CesDof29* at Chr32 all have one *Rhynchospora breviuscula* collinearity genes. These *C.esculentus* genes are part of the one-to-one collinearity relationship (Fig. 9D).

Figure 9E shows the 20 chromosomes of *Carex littledalei* (Chr1-Chr20) and the chromosomes of *C.esculentus*(Chr3-Chr5, Chr9, Chr12, Chr14, Chr16, Chr17, Chr27, Chr28, Chr30-Chr33, Chr36, Chr42, Chr50, and Chr54). The grey lines represent all the collinearity gene pairs of *Carex littledalei* and *C.esculentus*, while the red lines represent the *Dof* gene. The study found that *Dof* members from *Carex littledalei* and *C.esculentus* formed a total of 31 co-linear gene pairs. Co-linear gene pairs were identified on each chromosome pair of *C.esculentus*, with the highest number of co-linear gene pairs on chromosomes Chr6, Chr14, and Chr36, all of which had three co-linear pairs. *Carex littledalei* has collinearity gene pairs on all chromosomes except Chr8, Chr9, Chr12, Chr14, Chr16, and Chr19. The chromosome with the most collinearity gene pairs is Chr5 with 8 pairs, while Chr3, Chr5, Chr12, Chr27, Chr28, Chr30, Chr32, Chr42, Chr50 and Chr54 have only 1 collinearity gene pair. *CesDof04* on chromosome Chr33, *CesDof05* on chromosome Chr5, *CesDof09* on chromosome Chr17, *CesDof12* on chromosome Chr36, *CesDof15* on chromosome Chr6, *CesDof23* on chromosome Chr16 and *CesDof27* on chromosome Chr31 have two homozygous genes on *Carex littledalei*. The genes *CesDof01, CesDof03, CesDof06, CesDof07, CesDof10, CesDof11, CesDof13, CesDof14, CesDof16, CesDof17-CesDof19, CesDof20-CesDof21, CesDof22, CesDof25* and *CesDof29* each have only one corresponding collinearity *Carex littledalei* gene. These genes exhibit one-to-one collinearity and are located on various chromosomes including Chr30, Chr42, Chr12, Chr28, Chr3, Chr36, Chr27, Chr6, Chr5, Chr14, Chr9, Chr54, Chr50, and Chr32 (Fig. 9E).

**Fig. 10:**
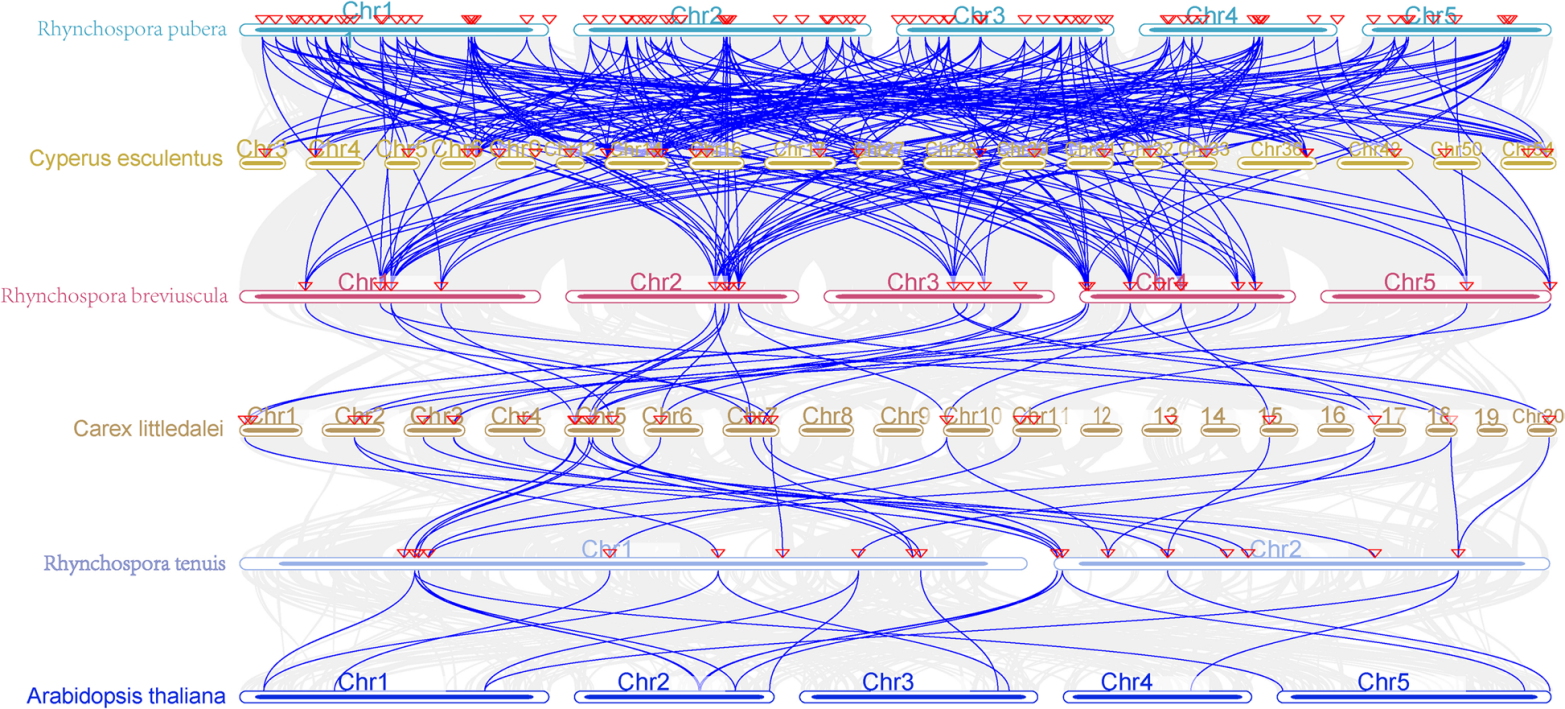
Colinearity relationship analysis between* Dof *genes in* C.esculentus *and other 5 species

Figure 10 shows the collinearity maps constructed of the same family between *C.esculentus*, *Arabidopsis thaliana* and four other species. The following collinearity relationships were obtained for the six species: 129 pairs of homologous genes between *C.esculentus* and *Rhynchospora pubera,* 114 genes in *Rhynchospora pubera* were in collinearity with *Rhynchospora breviuscula*, and 31 genes in *Rhynchospora breviuscula* are collinearity with *Carex littledalei* genes. There are 29 pairs of homologous gene pairs between *Carex littledalei* and *Rhynchospora tenuis,* as well as 18 pairs of collinearity gene pairs between *Rhynchospora tenuis* and *Arabidopsis thaliana*. These findings suggest that all six species share a common ancestor, with greater collinearity observed between plants of the same family (Fig. 10).

### Analysis of SSR loci in *CesDof* genes

The SSR loci analysis shows that the *CesDof* gene or promoter contained a total of 22 SSR sites, which were classified into 5 types: p1, p2, p3, p6, and c. Among them, p1 had 10 types, p2 had 4 types, and both p3 and p6 had only 1 type each, namely *CesDof10* and *CesDof13*, respectively. Additionally, there were 6 types of c. The SSR length of *CesDof22* was the longest at 103, while *CesDof16* had the shortest SSR length at 10(Supplementary Table 5).

### MiRNAs prediction of *CesDof* genes

These miRNAs of *Dof* genes have the function of regulating gene expression, therefore we predicted candidate miRNAs targeting the *CesDof* genes. It is expected that 144 miRNAs will target 26 *CesDof* genes. Among them, ath-miR5021 is predicted to target the most *CesDof* genes, with a total of 7, namely *CesDof08, CesDof09, CesDof10, CesDof15, CesDof14, CesDof19* and *CesDof26*; Next is ath-miR5014a-3p, which is predicted to target 5 *CesDof* genes, namely *CesDof04, CesDof08, CesDof10, CesDof15, CesDof27*. Other miRNAs are predicted to target one or different types of *CesDof* genes. Predicting 22 different miRNAs as target genes for the *CesDof08* gene; Next is the *CesDof15* gene, which has 19 different target genes for miRNAs. *CesDof04* and *CesDof21* genes have the least number, with only one target gene for different miRNAs, namely ath-miR5014a-3p and ath-miR1888a(Supplementary Table 6).

### Protein–protein interactions of CesDof proteins

**Fig. 11:**
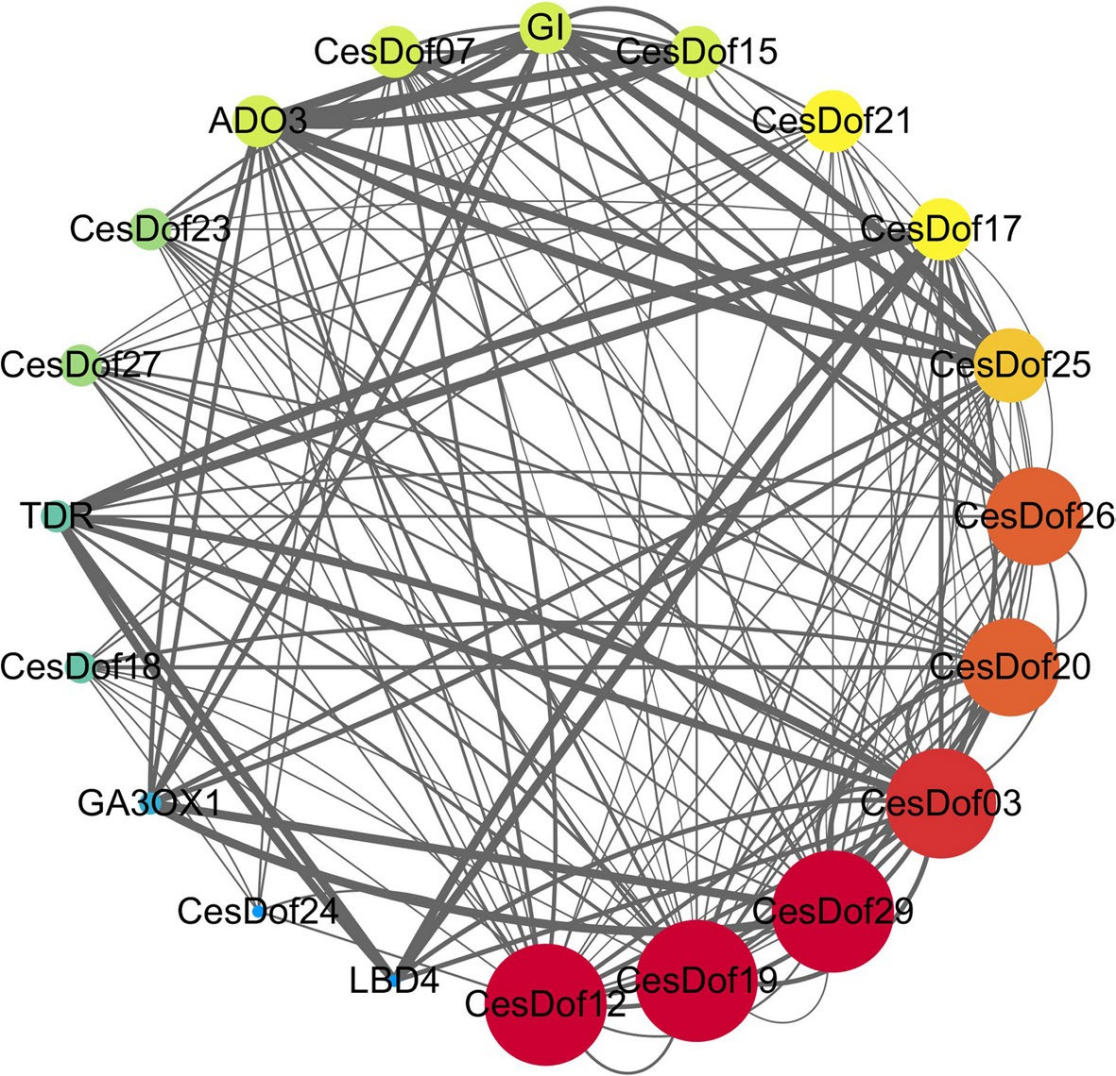
Interaction protein network of CesDof proteins

Protein–protein interaction analysis showed protein network of CesDof protein interactions was constructed by using the STRING online database. The results indicate an interaction relationship between the proteins of *GI, ADO3, TDR, GA3OX1, LBD4, CesDof17, CesDof27, CesDof23, CesDof15, CesDof07, CesDof34, CesDof18, CesDof20, CesDof26, CesDof25, CesDof12, CesDof03, CesDof29, CesDof19* and *CesDof21*. The diagram shows the interaction network of functional genes, with lines connecting them and the thickness of the lines indicating the strength of the interaction. The size and colour depth of the nodes indicate that *CesDof12, CesDof03, CesDof29, CesDof19, GI* and *ADO3* are the core proteins. *CesDof12* interacts with 13 proteins, while *CesDof03* and *CesDof29* interact with 12 proteins each. *CesDof19, GI* and *ADO3* have 10, 7, and 7 interacting proteins, respectively (Fig. 11).

### Gene expression analysis of *CesDof* gene family under drought stress and salt stress conditions by qRT-PCR

**Fig. 12:**
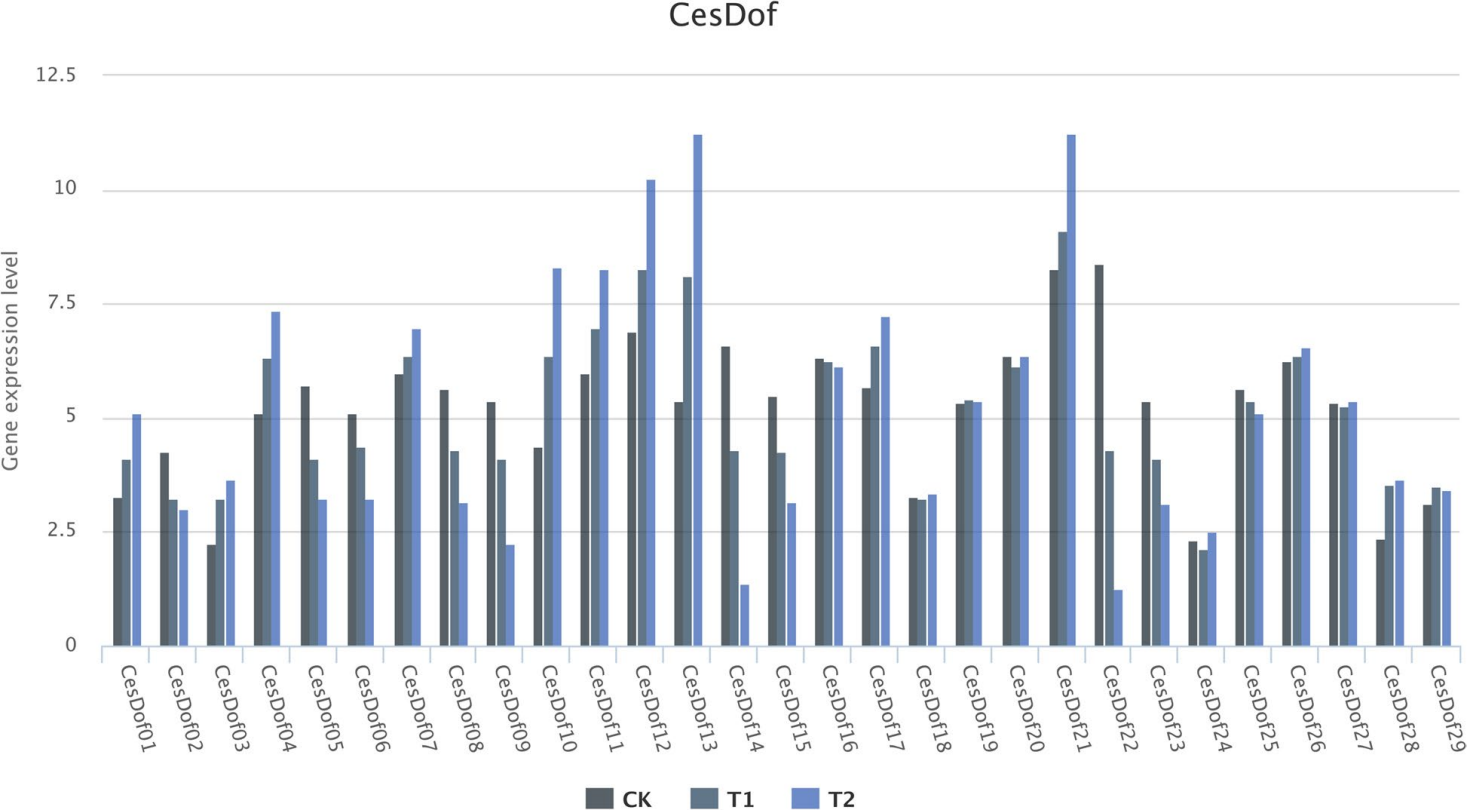
Gene expression level of CesDof under drought stress by qRT-PCR

Through qRT-PCR experiments on *CesDof* gene family in the leaves of *C.esculentus* seedlings after 7 days and 14 days of drought treatment, the results showed that compared with the control group (CK), gene expression level of 11 *CesDof* genes (*CesDof01, CesDof03, CesDof04, CesDof07, CesDof10, CesDof11, CesDof12, CesDof13, CesDof17, CesDof21, CesDof28*) in *C.esculentus* in the drought treatment group (T) were higher than those in the control group. The expression levels of 9 *CesDof* genes in the drought treatment group (T), including *CesDof02, CesDof05, CesDof06, CesDof08, CesDof09, CesDof14, CesDof15, CesDof22* and *CesDof23* were lower than those of the control group. The remaining 9 *CesDof* genes in this gene family showed little change in expression content after drought stress treatment. These results suggest that most *CesDof* genes are very responsive to drought stress, indicating that most *CesDof* genes may be involved in drought stress responses (Fig. 12).

**Fig. 13:**
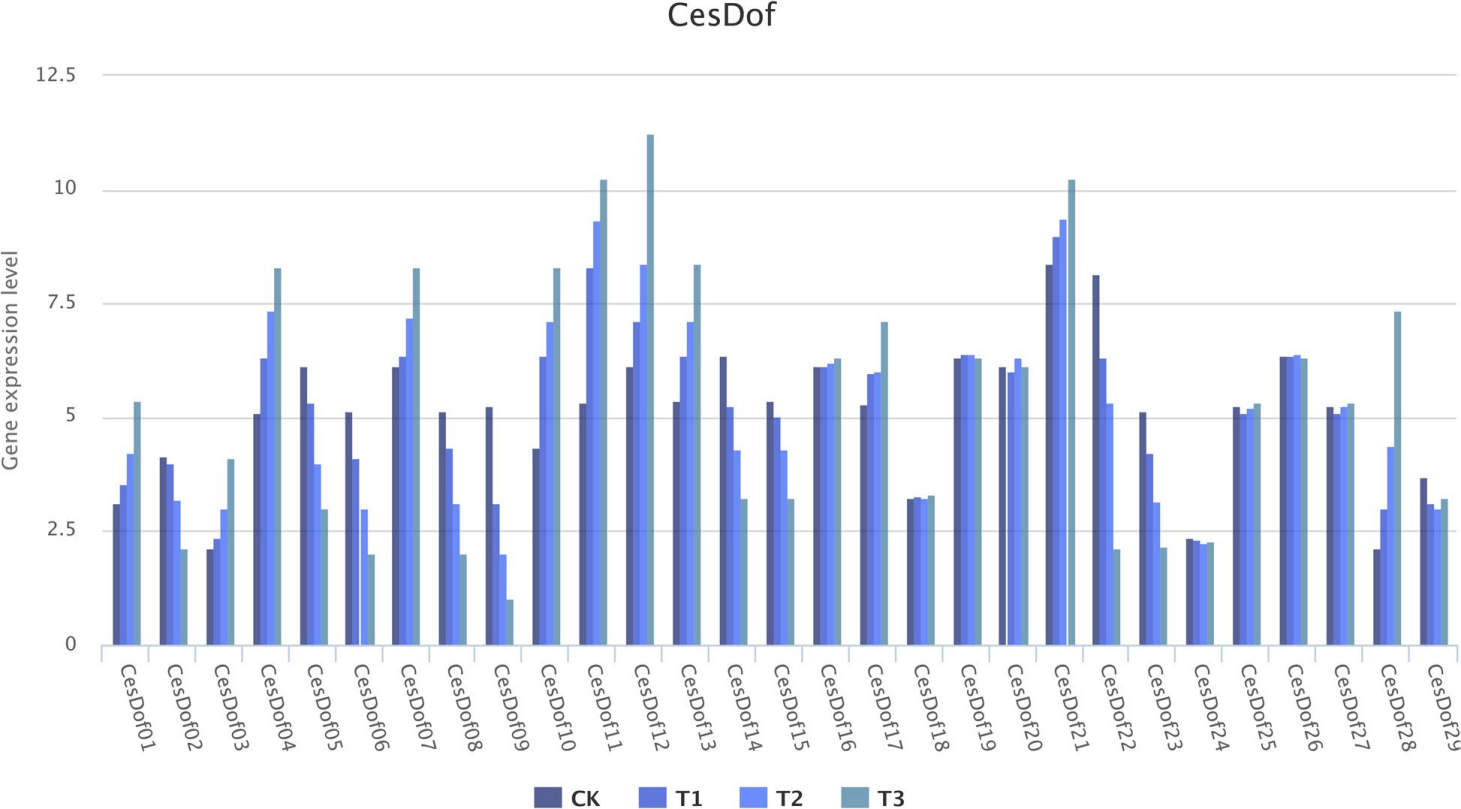
Gene expression level of CesDof under salt stress by qRT-PCR

The qRT-PCR analysis of the salt stress treatment experiment showed that compared with the control group, the gene expression levels of 11 *CesDof* genes increased with increasing NaCl concentration, namely *CesDof01, CesDof03, CesDof04, CesDof07, CesDof10, CesDof11, CesDof12, CesDof13, CesDof17, CesDof21, CesDof28*. The expression levels of 9 *CesDof* genes decreased, namely *CesDof02, CesDof05, CesDof06, CesDof08, CesDof09, CesDof14, CesDof15, CesDof22* and *CesDof23*, and only the remaining 9 *CesDof* genes showed little change in expression levels. These results indicate that most *CesDof* genes have a relatively large response to salt stress, and suggest that *CesDof* gene family may be involved in their salt stress response (Fig. 13). We were pleasantly surprised to find that the mechanisms of *CesDof* gene family in response to drought and salt stress are similar, and the change trends of gene expression levels of many *CesDof* genes in response to drought and salt stress are consistent.

## Discussion

*Dof* gene family is a plant-specific transcription factor that plays a crucial role in various physiological processes, including plant growth and development. Currently, *Dof* gene family of plant genomes has been identified in various plants, such as rice, wheat, maize, and tobacco. However, there are no relevant reports on the whole genome of the *Dof* gene family in the perennial herbaceous plant *C.esculentus*. In this study, we identified a total of 29 *Dof* transcription factors in *C.esculentus*, which is a similar number to those found in rice (30), tomato (34) and lotus (29). We analyzed their physicochemical properties, chromosomal localization, and secondary and tertiary structures. The chromosome localization was also examined. The *Dof* gene family of *C.esculentus* was analyzed for various aspects including secondary and tertiary structures, phosphorylation sites, hydrophilicity and hydrophobicity, conserved motifs, cis-acting elements, transcription factor binding sites, phylogenetic tree analysis, collinearity analysis, SSR analysis, miRNA prediction, and protein interaction.

Analysis of structural domains and motifs of 29 *Dof* gene families in *C.esculentus* revealed that they all contain complete C2-C2 single finger zinc structures, which is consistent with the studies of Zou [4], Li [9]. Different gene families have unique motifs, and *CesDof* has a common motif in *C.esculentus*, indicating that the conserved motif plays an important role. In addition, the diversity of motifs between different *CesDof* may be related to their complex functions.

Within the 29 family members, the majority of *C.esculentus Dof* gene family either lack introns or have only one. This indicates that the *Dof* gene structure is conserved, which is consistent with the gene structure reported in lotus [38], wheat [12], rice [39], and tomato [40].

The isoelectric point (pI) value of *C.esculentus DOF* family ranges from 4.71 to 9.84, according to the physicochemical properties of the *C.esculentus DOF* protein. This is similar to the results predicted by scientists who identified the tobacco *Dof* gene in the common tobacco *K326* genome [41] and *DOF* gene in the annual alfalfa identification [42]. Most members are basic and unstable proteins, which is consistent with the research structure of Sunshouru and other scholars on American pumpkin [43]. All 29 members were hydrophilic, non-secretory proteins, and located in the nucleus, indicating that the *DOF* protein plays a regulatory role in plant growth and development. This positioning is consistent with the prediction result of Rose [44].

The analysis of amino acid transmembrane structure, hydrophobicity, and phosphorylation sites of *DOF* family members indicates that the members of *DOF* family in *C.esculentus* are hydrophilic proteins and lack transmembrane domains. Their protein functions are mainly realized through phosphorylation at serine sites. These results are consistent with those predicted by scholars in Brassica napus [45]. The secondary structure prediction shows that *C.esculentus DOF* family members are primarily composed of random coils, α-helix, extended chain structures, and relatively small amounts of β-turns, with the lowest occurrence of β-turns. The description of β-turns and extension chains is scattered throughout the entire amino acid. The online tool MEME was used to analyze 29 *Dof* genes of *C.esculentus*. This resulted in the identification of 10 independent conserved motifs. The core component of *C.esculentus DOF* protein is Motif1, which contains a single zinc finger structure (C2-C2). This motif is present in all 29 family members, which is consistent with research on the transcription factor of linseed mustard *Dof* [46]. These results suggest that Dof proteins with the same conserved motifs in the same group may have the same function.

By analyzing the promoter region of 2000 bp upstream of *CesDof* sequence, it was found that they may play a role in hormone response, light response, growth and development, and the light response element is one of the typical characteristic elements. Previous studies have shown that in Arabidopsis, if the *DOF* of OBP strain is highly expressed, the light response elements will change, and then affect the growth of *Arabidopsis* [47]. In *Phyllostachys edulis, PheDof12-1* can regulate photoperiod related regulatory factors when put *PheDof12-1* gene into different environments, such as cold, drought, salt and gibberellin (GA3). Through ectopic expression in Arabidopsis, it was found that the overexpressed homozygous *PheDof12-1* of transgenic Arabidopsis showed early flowering under long day (LD) conditions, combined with the promoter sequence of *PheCOL4*, and had a strong circadian pattern, These results indicate that *DOF* transcription factor is involved in regulating the flowering time of *Phyllostachys heterocycla* [48]. This suggests that the members of the *DOF* family may be closely related to the light regulatory elements. In addition, *DOF* also affects the regulation of plant growth and development by regulating the expression of a variety of hormones. For example, the *DOF* transcription factor ntbbf1 (rolB domain B factor 1) in *Nicotiana tabacum* binds to the acttta region of the gene *rolB* promoter, thereby regulating the tissue-specific expression of *rolB* gene and the growth factor induced expression [49], promoting root growth [50]. The analysis of cis element of promoter will lay a foundation for further study of the potential function of *CesDof* involved in light signal and hormone related pathways. The *Dof* genes may play an important role in plant growth, light response, hormone response and stress response. This is consistent with the previous studies that the *Dof* gene family responds to light and participates in metabolism.

Further study of transcription binding factors identified 44 types in 29 *CesDof*, including *Dof, ERF, MYB, BBR-BPC, MIKC_MADS.*etc. This suggests that various transcription binding factors play a crucial role in the growth, development, reproduction, and other physiological processes of *C.esculentus*, and they must coordinate with each other. Research has shown that the *DOF* protein is a multifunctional transcription factor that affects various aspects of plant growth and development, including light response, tissue differentiation, seed development or germination, and plant hormone signal transduction [51]. For instance, when rice highly expresses *OsDof04*, it can respond to light and affect flowering time depending on the duration of light exposure [52]. Apple *MdDof54* regulates the expression of drought response genes, root development, and photosynthesis, affecting drought resistance. It also shows a positive correlation [53]. Overexpression of *PhDof28* in *Petunia hybrida* promotes the accumulation of IAA, resulting in petal elongation [54].

The codon preference of 29 *C.esculentus DOF* genes was investigated. It was found that the gene preference of *C.esculentus* ended with T/A, and AGA (RSCU = 1.48) was the 1 synonymous codon with the strongest preference for *C.esculentus*. AUG, AUA, GGC and UGG had no codon preference (RSCU = 1), while others were less than 1. The ENC ranged from 41.31 to 57.81, with an average of 52.93. The CAI value ranged from 0.135 to 0.275. This is similar to the research findings of the strawberry DHAR gene preference [55] and the Siberian apricot pssoc1-like gene codon preference [56]. These results indicate that the *C.esculentus* gene has a weak codon preference and a low transcription expression level.

MIRNAs are small endogenous RNAs that exist widely in plants and function in post-transcriptional regulation of gene expression. They play an important role in plant growth, development, and abiotic stress response [57]. In bananas, 30 miRNAs are estimated to target 12 mafad members, with ac-mir156 and mac-mir164 targeting the largest number of genes [58]. 19 miRNAs are predicted to target twenty ahfads in peanuts, including four FAD3s, four FAD2s, two FAD7s, one FAD8, and eight SLDS [59]. The study discovered that 144 miRNAs were expected to target 29 *CesDof* genes, with ath-mir5021 targeting seven different *CesDof* genes. It is possible that these miRNAs directly regulate the expression of these *CesDof* genes, but this requires confirmation in future studies.

The phylogenetic tree for the *Dof* genes of *C.esculentus* and other plants was constructed by the author. The results indicate that the 29 *Dof* genes of *C.esculentus* were divided into four categories based on their phylogenetic relationship and sequence similarity. This finding is consistent with previous research on *Dof* gene family of Arabidopsis, rice and wheat [8, 60–62]. Most of the *CesDof* genes within the same subfamily exhibit similar motifs, suggesting that these conserved motifs may be crucial for group or subgroup-specific functions. The distribution of motifs among different subfamilies suggests the complexity of protein function in the *DOF* gene family of *C.esculentus*. However, the number of introns in *CesDof* genes was mostly between 0 and 2, indicating evolutionary conservation. There were no significant differences between the exons and introns of *CesDof* gene family members. The phylogenetic tree of 29 *CesDof* genes and *AthDof* genes was constructed by the author, which were roughly divided into five groups. *CesDof* genes were significantly reduced in the process of plant evolution, leaving only *AthDof* genes in group1 and only *CesDof* genes in Group9. Therefore, it is speculated that there are differences in the evolution of *DOF* genes in different species. The phylogenetic analysis of *Dof* gene family in *C.esculentus*, *Cyperaceae*, *Arabidopsis thalianaand* other species revealed that *Dof* gene family tree in *C.esculentus* and six other plants was divided into ten groups, namely group 1 to group 10. CesDof protein members were primarily distributed in Group 4, 7, and 10, with some presence in Group 3 and 9. Group 6 and 8 did not contain any CesDof protein members, indicating that the homologous genes of *CesDof* in these two groups may have been lost during the evolutionary process.

To investigate the origin and evolution of *Dof* gene of *C.esculentus*, we conducted a collinearity analysis of the *Dof* gene families of *C.esculentus*, *Arabidopsis thaliana,* and other *Cyperaceae* plants. The analysis revealed three pairs of homologous genes among the 29 *CesDof* genes. Furthermore, most of *CesDof* genes did not have any homologous gene members on chromosomes. The uneven distribution of *CesDof* gene family during chromosome evolution may explain this phenomenon. Collinearity analysis revealed that 15 out of 29 *CesDof* genes were collinear with *Arabidopsis thaliana.* Both *CesDof18* and *CesDof14* had three homologous genes, indicating a relatively close genetic relationship between *Arabidopsis thaliana* and *C.esculentus*. The *Dof* gene has been conserved throughout the process of plant evolution, even among different species. In *C.esculentus*, many genes in *Arabidopsis* have more than two homologous genes, indicating a shared origin between the two. Thirty-one of *Carex littledalei* genes were collinear with *CesDof*, and 79% of *CesDof* had 1–2 homologous genes in *Carex littledalei*. Twenty-eight of the genes were collinear with *Rhynchospora tenuis*, representing 72% of the total. Additionally, 33 genes were collinear with *Rhynchospora breviuscula*, accounting for 76% of the total. There was a colinearity relationship between 129 *Rhynchospora pubera* genes and *CesDo*f genes. Further statistical analysis of the homologues of *Dof* genes in *Rhynchospora pubera* in *C.esculentus* revealed that 83% of *CesDof* genes had homologues in *Rhynchospora pubera*. Among them, *CesDof19* had six homologous genes in *Rhynchospora pubera*. This suggests that the amplification of *Dof* gene family may have occurred prior to the differentiation of *C.esculentus* and *Rhynchospora pubera*. This indicates that *Dof* gene is more conserved among plants of the same family, and that the genetic relationship between *C.esculentus* and *Rhynchospora pubera* is much closer than that of other species. The collinearity relationship of *C.esculentus* with five other species indicates the following relationships: *C.esculentus*, *Rhynchospora pubera, Rhynchospora breviuscula*, Carex littledalei*, Rhynchospora tenuis* and *Arabidopsis thaliana*. The closest affinity is between *C.esculentus* and *Rhynchospora pubera,* indicating a closer evolutionary relationship. On the other hand, *C.esculentus* and *Arabidopsis thaliana* have the furthest affinity, suggesting that *Dof* plants are more conserved among conspecifics. Conversely, *C.esculentus* and *Arabidopsis thaliana* have the farthest genetic relationship. The analysis suggests that *Dof* plants are more conservative among the same family of plants. From a plant taxonomy perspective, *C.esculentus* and *Carex littledalei, Rhynchospora tenuis* and *Rhynchospora breviuscula* are both *Cyperaceae* plants with a close genetic relationship. The number of homologous gene pairs between *Cyperaceae* plants is greater than that between *C.esculentus* and *Arabidopsis,* indicating a closer genetic relationship.

To identify the selection pressure of the *CesDof* repeat gene in the evolution process of *C.esculentus*, three *CesDof* repeat events were analyzed by Ka/Ks. If the Ka/Ks ratio is greater than 1, it is considered that *CesDof* gene has undergone a positive selection effect in the evolutionary process, accelerating its evolution. When the Ka/Ks ratio is equal to 1, neutral selection is considered to be acting on the *CesDof* gene, meaning that natural selection does not affect its evolution. If the Ka/Ks ratio is less than 1, it is considered that the *CesDof* gene has undergone purifying selection [63]. It was found that the Ka/Ks values of the three *CesDof* repeat events ranged from 0.21 (*CesDof22*/*CesDof29*) to 0.35 (*CesDof24*/*CesDof12)*, and the ratio was less than 1. Therefore, the *CesDof* repeat gene was selected during the process of evolution.

Dof proteins have been shown to interact with other proteins [64, 65]. Interacting protein prediction analyzes indicate that some of *C. esculentus Dof* family proteins have close interactions with each other. The results indicate that 15 *Dof* proteins interact with *Arabidopsis GI* protein, *ADO3* protein, *TDR* protein, *GA3OX1* protein and *LBD4* protein. *Cesdof12* and *CesDof29* are homologous to zinc finger proteins *Dof2.5* and *Dof3.7* of *Arabidopsis*, respectively. They play a role in maternal control of seed germination and regulate transcription by binding to the common core sequence of 5'-AA[Ag]G-3'. *Cesdof03* is a homologous protein to *Arabidopsis* zinc finger protein *Dof5.6*. It specifically binds to the 5'-AA[Ag]G-3' consensus core sequence, promotes the radial growth of protophloem sieve elements, and participates in the formation of interfascicular cambium and the regulation of vascular tissue development, particularly in the early stage of inflorescence stem development. *Cesdof19* and the zinc finger protein *Dof5.1* of *Arabidopsis thaliana* are homologous and can specifically bind to the 5'-AA[AG]G-3' consensus core sequence. They also bind to the 5'-TAAAGT-3' motif in the *Rev* promoter to trigger its transcription, thereby regulating paraxial polarity and auxin transport, including the expression of IAA6 and IAA19 genes. Gigantea (GI) is a plant-specific circadian clock control gene that interacts with CONSTANS (CO) to regulate the long-day flowering pathway. It primarily regulates photoperiodic flowering and the circadian rhythm of plants [66]. This protein has a pleiotropic function in the entire developmental stage, from seed germination to flowering time control [67], and also participates in red light signals and controls circadian clock function. The *SCF (ADO3) E3* ubiquitin ligase complex regulates circadian rhythm and has diverse roles. Some core proteins are involved in seed germination, others in circadian rhythm and photoperiod regulation, and some in the radial growth of protophloem sieve elements. These proteins form complexes with *DOF* and other types of proteins through direct protein–protein interactions, cooperating to regulate the growth of *C.esculentus*.

## Conclusion

In this study, we have identified 29 *CesDof* genes in *C.esculentus* genome. They are located in the nucleus and have a range of 124 to 512 amino acids, with most being basic proteins. Their secondary structure is mainly random coil. The promoter sequence of *CesDof* genes contains cis-acting elements that respond to light, drought, hormones, low temperature, and circadian rhythm. The *CesDof* genes' codon preference ends in T/A. *C.esculentus* had three pairs of collinear *CesDof* genes. Additionally, there were 15 pairs of collinear genes shared between *C.esculentus* and *Arabidopsis thaliana.* The genetic relationship between *C.esculentus* and *Rhynchospora pubera* was found to be the closest. Phylogenetic tree analysis revealed that the 29 *CesDof* genes of *C.esculentus* can be classified into 4 subgroups. Additionally, 144 miRNAs were predicted to target these *CesDof* genes. Furthermore, protein interaction analysis indicated that 15 *Dof* proteins in *C.esculentus* have interactions. Most *CesDof* genes were involved in drought stress and salt stress responses, and the gene expression trends under drought stress and salt stress conditions were consistent These results lay a theoretical foundation for further studying the molecular functions of *Dof* gene family in *C.esculentus* and its molecular mechanisms in regulating the life activities of *C.esculentus.*

### Supplementary Information

Supplementary Material 1.

Supplementary Material 2.

Supplementary Material 3.

Supplementary Material 4.

## Data Availability

The whole genome sequence, protein sequence, and gene annotation files of C.esculentus were downloaded from China National GeneBank DataBase (CNGBdb)(https://ftp.cngb.org/pub/CNSA/data5/CNP0003839/CNS0648185/CNA0051961/)
