# Supplementary material for: Genome-wide identification and molecular evolution of Dof transcription factors in Cyperus esculentus
Source: BMC Genomics. 2024 Jul 3;25:667. doi: 10.1186/s12864-024-10565-y (PMC11223408; doi:10.1186/s12864-024-10565-y)
Supplement: Supplementary file 2 — Supplementary Material 2. [file 12864_2024_10565_MOESM2_ESM.docx]

Supplementary Table2 Secondary structure analysis of Dof protein from *C.esculentus*

| Protein | Alpha helix/% | Extended strand/% | Beta turn/% | Random coil/% |
| --- | --- | --- | --- | --- |
| *CesDof01* | 19.53 | 8.2 | 2.93 | 69.34 |
| *CesDof02* | 10.56 | 16.67 | 7.22 | 65.56 |
| *CesDof03* | 18.79 | 16.31 | 3.19 | 61.7 |
| *CesDof04* | 10.65 | 15.48 | 3.23 | 70.65 |
| *CesDof05* | 9.70 | 18.33 | 8.89 | 63.07 |
| *CesDof06* | 27.64 | 8.04 | 5.53 | 58.79 |
| *CesDof07* | 17.06 | 11.47 | 3.24 | 68.24 |
| *CesDof08* | 10.02 | 16.45 | 1.97 | 71.38 |
| *CesDof09* | 8.66 | 14.57 | 5.51 | 71.26 |
| *CesDof10* | 10.51 | 9.31 | 1.80 | 78.38 |
| *CesDof11* | 9.14 | 91.4 | 1.71 | 80.00 |
| *CesDof12* | 12.97 | 9.9 | 4.10 | 73.04 |
| *CesDof13* | 22.61 | 15.71 | 4.21 | 57.47 |
| *CesDof14* | 12.90 | 9.05 | 2.26 | 75.79 |
| *CesDof15* | 12.05 | 8.26 | 1.56 | 78.12 |
| *CesDof16* | 13.07 | 19.1 | 6.03 | 61.81 |
| *CesDof17* | 11.11 | 12.15 | 2.08 | 74.65 |
| *CesDof18* | 15.69 | 18.61 | 4.74 | 60.95 |
| *CesDof19* | 15.00 | 10.71 | 3.57 | 70.71 |
| *CesDof20* | 11.07 | 17.34 | 2.95 | 68.63 |
| *CesDof21* | 14.22 | 17.54 | 6.64 | 61.61 |
| *CesDof22* | 18.34 | 12.66 | 4.80 | 64.19 |
| *CesDof023* | 14.80 | 13.90 | 5.83 | 65.47 |
| *CesDof024* | 28.97 | 6.21 | 5.52 | 59.31 |
| *CesDof025* | 12.90 | 12.10 | 4.03 | 70.97 |
| *CesDof026* | 13.33 | 11.23 | 10.18 | 65.26 |
| *CesDof027* | 8.72 | 8.14 | 4.65 | 78.49 |
| *CesDof028* | 6.55 | 10.12 | 1.19 | 82.14 |
| *CesDof029* | 11.26 | 12.61 | 4.50 | 71.62 |

Supplementary Table 3 *T3s，C3s，A3s，G3s，CAI，CBI，Fop，Nc* and *GC* of *Dof* gene family in *C.esculentus*

| genome | *T3s* | *C3s* | *A3s* | *G3s* | *CAI* | *CBI* | *Fop* | *Nc* | *GC3s* | *GC* |
| --- | --- | --- | --- | --- | --- | --- | --- | --- | --- | --- |
| *CesDof1* | 0.3276 | 0.3405 | 0.2438 | 0.2961 | 0.184 | 0.011 | 0.42 | 57.24 | 0.514 | 0.508 |
| *CesDof2* | 0.2547 | 0.4151 | 0.1605 | 0.4487 | 0.262 | 0.155 | 0.521 | 54.67 | 0.664 | 0.554 |
| *CesDof3* | 0.2908 | 0.3426 | 0.1302 | 0.419 | 0.221 | 0.133 | 0.498 | 49.71 | 0.633 | 0.608 |
| *CesDof4* | 0.1421 | 0.4315 | 0.2118 | 0.4364 | 0.247 | 0.218 | 0.548 | 52.04 | 0.71 | 0.587 |
| *CesDof5* | 0.2919 | 0.3478 | 0.2414 | 0.2824 | 0.185 | 0.147 | 0.474 | 53.7 | 0.531 | 0.552 |
| *CesDof6* | 0.2462 | 0.4269 | 0.2186 | 0.3564 | 0.233 | 0.17 | 0.517 | 51.05 | 0.622 | 0.53 |
| *CesDof7* | 0.3704 | 0.2634 | 0.2489 | 0.2884 | 0.163 | 0.073 | 0.44 | 45.02 | 0.458 | 0.51 |
| *CesDof8* | 0.3813 | 0.2848 | 0.3757 | 0.2441 | 0.186 | -0.103 | 0.355 | 52.32 | 0.406 | 0.416 |
| *CesDof9* | 0.4202 | 0.2821 | 0.3521 | 0.2333 | 0.186 | -0.089 | 0.364 | 52.7 | 0.39 | 0.395 |
| *CesDof10* | 0.3954 | 0.2949 | 0.3661 | 0.1934 | 0.178 | -0.006 | 0.409 | 52.67 | 0.382 | 0.4 |
| *CesDof11* | 0.2283 | 0.3533 | 0.2601 | 0.3434 | 0.135 | -0.041 | 0.368 | 57.26 | 0.584 | 0.602 |
| *CesDof12* | 0.3566 | 0.2746 | 0.242 | 0.3092 | 0.179 | 0.031 | 0.413 | 54.77 | 0.483 | 0.534 |
| *CesDof13* | 0.3274 | 0.3274 | 0.1925 | 0.3309 | 0.213 | 0.104 | 0.471 | 54.18 | 0.54 | 0.524 |
| *CesDof14* | 0.4079 | 0.296 | 0.3293 | 0.24 | 0.213 | -0.032 | 0.409 | 56.97 | 0.413 | 0.412 |
| *CesDof15* | 0.4415 | 0.245 | 0.3885 | 0.1788 | 0.18 | -0.097 | 0.356 | 52.22 | 0.331 | 0.362 |
| *CesDof16* | 0.396 | 0.2409 | 0.4148 | 0.2174 | 0.171 | -0.072 | 0.364 | 54.51 | 0.351 | 0.386 |
| *CesDof17* | 0.385 | 0.2676 | 0.3621 | 0.2651 | 0.172 | -0.1 | 0.37 | 52.4 | 0.411 | 0.44 |
| *CesDof18* | 0.3152 | 0.2939 | 0.3376 | 0.2552 | 0.153 | 0.014 | 0.424 | 48.75 | 0.45 | 0.488 |
| *CesDof19* | 0.2905 | 0.4022 | 0.2763 | 0.242 | 0.191 | 0.038 | 0.433 | 54.92 | 0.53 | 0.524 |
| *CesDof20* | 0.3208 | 0.2594 | 0.3279 | 0.2865 | 0.19 | -0.092 | 0.353 | 48.64 | 0.448 | 0.467 |
| *CesDof21* | 0.2683 | 0.3798 | 0.2023 | 0.3548 | 0.254 | 0.155 | 0.528 | 55.78 | 0.604 | 0.565 |
| *CesDof22* | 0.3184 | 0.3245 | 0.3757 | 0.2206 | 0.172 | -0.003 | 0.417 | 53.64 | 0.433 | 0.467 |
| *CesDof23* | 0.4012 | 0.2765 | 0.3375 | 0.2658 | 0.19 | -0.068 | 0.376 | 53.85 | 0.411 | 0.42 |
| *CesDof24* | 0.4377 | 0.2494 | 0.3479 | 0.2421 | 0.223 | 0.075 | 0.454 | 53.65 | 0.369 | 0.395 |
| *CesDof25* | 0.1429 | 0.585 | 0.1418 | 0.3282 | 0.267 | 0.304 | 0.598 | 41.31 | 0.763 | 0.671 |
| *CesDof26* | 0.3459 | 0.2432 | 0.3856 | 0.2572 | 0.171 | -0.117 | 0.332 | 57.81 | 0.396 | 0.425 |
| *CesDof27* | 0.3767 | 0.3008 | 0.3425 | 0.2432 | 0.201 | -0.021 | 0.409 | 56.94 | 0.421 | 0.428 |
| *CesDof28* | 0.3743 | 0.3583 | 0.2339 | 0.2123 | 0.228 | 0.24 | 0.538 | 50.28 | 0.471 | 0.48 |
| *CesDof29* | 0.3098 | 0.3587 | 0.25 | 0.3382 | 0.275 | 0.15 | 0.529 | 56 | 0.549 | 0.521 |

Supplementary Table4 Relative Frequency of Synonymous Codon of *Dof* gene family in *C.esculentus*

| amino acid | codon | number | RSCU | amino acid | codon | number | RSCU |
| --- | --- | --- | --- | --- | --- | --- | --- |
| Phe | UUU | 408 | 1.21 | Ser | UCU | 237 | 1.25 |
|  | UUC | 266 | 0.79 |  | UCC | 201 | 1.06 |
| Leu | UUA | 225 | 1.05 |  | UCA | 232 | 1.23 |
|  | UUG | 290 | 1.36 |  | UCG | 127 | 0.67 |
|  | CUU | 268 | 1.26 | Pro | CCU | 147 | 0.9 |
|  | CUC | 174 | 0.81 |  | CCC | 132 | 0.81 |
|  | CUA | 112 | 0.52 |  | CCA | 203 | 1.24 |
|  | CUG | 211 | 0.99 |  | CCG | 171 | 1.05 |
| Ile | AUU | 299 | 1.28 | Thr | ACU | 174 | 1.18 |
|  | AUC | 170 | 0.73 |  | ACC | 158 | 1.07 |
|  | AUA | 234 | 1 |  | ACA | 185 | 1.26 |
| Met | AUG | 198 | 1 |  | ACG | 71 | 0.48 |
| Val | GUU | 227 | 1.4 | Ala | GCU | 175 | 0.97 |
|  | GUC | 120 | 0.74 |  | GCC | 164 | 0.91 |
|  | GUA | 153 | 0.94 |  | GCA | 255 | 1.42 |
|  | GUG | 150 | 0.92 |  | GCG | 124 | 0.69 |
| Tyr | UAU | 248 | 1.18 | Cys | UGU | 244 | 0.98 |
|  | UAC | 173 | 0.82 |  | UGC | 254 | 1.02 |
| TER | UAA | 222 | 1.17 | TER | UGA | 220 | 1.16 |
|  | UAG | 127 | 0.67 | Trp | UGG | 226 | 1 |
| His | CAU | 208 | 1.03 | Arg | CGU | 68 | 0.48 |
|  | CAC | 194 | 0.97 |  | CGC | 124 | 0.86 |
| Gln | CAA | 320 | 1.16 |  | CGA | 115 | 0.79 |
|  | CAG | 230 | 0.84 |  | CGG | 184 | 1.27 |
| Asn | AAU | 303 | 1.15 | Ser | AGU | 172 | 0.91 |
|  | AAC | 224 | 0.85 |  | AGC | 165 | 0.87 |
| Lys | AAA | 351 | 1.21 | Arg | AGA | 214 | 1.48 |
|  | AAG | 229 | 0.79 |  | AGG | 162 | 1.12 |
| Asp | GAU | 181 | 1.18 | Gly | GGU | 173 | 1.1 |
|  | GAC | 126 | 0.82 |  | GGC | 157 | 1 |
| Glu | GAA | 219 | 1.13 |  | GGA | 161 | 1.03 |
|  | GAG | 170 | 0.87 |  | GGG | 136 | 0.87 |

Supplementary Table5 Predicted SSR loci of *Dof* gene family in *C.esculentus*

| gene name | SSR nr. | SSR type | SSR-- | SSR length |
| --- | --- | --- | --- | --- |
| *CesDof8* | 1 | p1 | (T)12 | 12 |
| *CesDof9* | 1 | c | (T)18cccttttcctttttgttatgtatatttttttgcttgttggt  ggttggtagttgtatcttgtgtgtaat(TA)6 | 98 |
| *CesDof9* | 2 | p1 | (T)14 | 14 |
| *CesDof10* | 1 | p3 | (TCC)6 | 18 |
| *CesDof10* | 2 | p2 | (CT)8 | 16 |
| *CesDof13* | 1 | p6 | (CCATCA)5 | 30 |
| *CesDof14* | 1 | c | (A)10ttgaggaacatattatgtataacatg(TA)6 | 48 |
| *CesDof15* | 1 | p1 | (A)13 | 13 |
| *CesDof15* | 2 | c | (T)10gttaagtttggtttctctccatggatctctacaagaag-tgtaaattttgttactgttttgttgtacaaatatttgga(T)15 | 102 |
| *CesDof15* | 3 | p1 | (T)13 | 13 |
| *CesDof15* | 4 | c | (T)16ctttttcatctctggtgagattctttcataaac(T)11 | 60 |
| *CesDof15* | 5 | p1 | (T)19 | 19 |
| *CesDof16* | 1 | p1 | (A)17 | 17 |
| *CesDof16* | 2 | p1 | (A)12 | 12 |
| *CesDof16* | 3 | p1 | (A)10 | 10 |
| *CesDof18* | 1 | c | (A)12g(A)10gtcggatcgaaaaaggagg(A)10 | 52 |
| *CesDof22* | 1 | p1 | (A)19 | 19 |
| *CesDof22* | 2 | c | (AACA)5tagatctacaagtttgctagctagctccaaagcag  ccatggcagcttcttcaggaaaaacccaaaaacc(A)14 | 103 |
| *CesDof23* | 1 | p2 | (TA)12 | 24 |
| *CesDof24* | 1 | p2 | (AT)19 | 38 |
| *CesDof26* | 1 | p1 | (T)22 | 22 |
| *CesDof26* | 2 | p2 | (AT)6 | 12 |
