# Supplementary material for: Genome-wide identification and molecular evolution of Dof transcription factors in Cyperus esculentus
Source: BMC Genomics. 2024 Jul 3;25:667. doi: 10.1186/s12864-024-10565-y (PMC11223408; doi:10.1186/s12864-024-10565-y)
Supplement: Supplementary file 4 — Supplementary Material 4. [file 12864_2024_10565_MOESM4_ESM.docx]

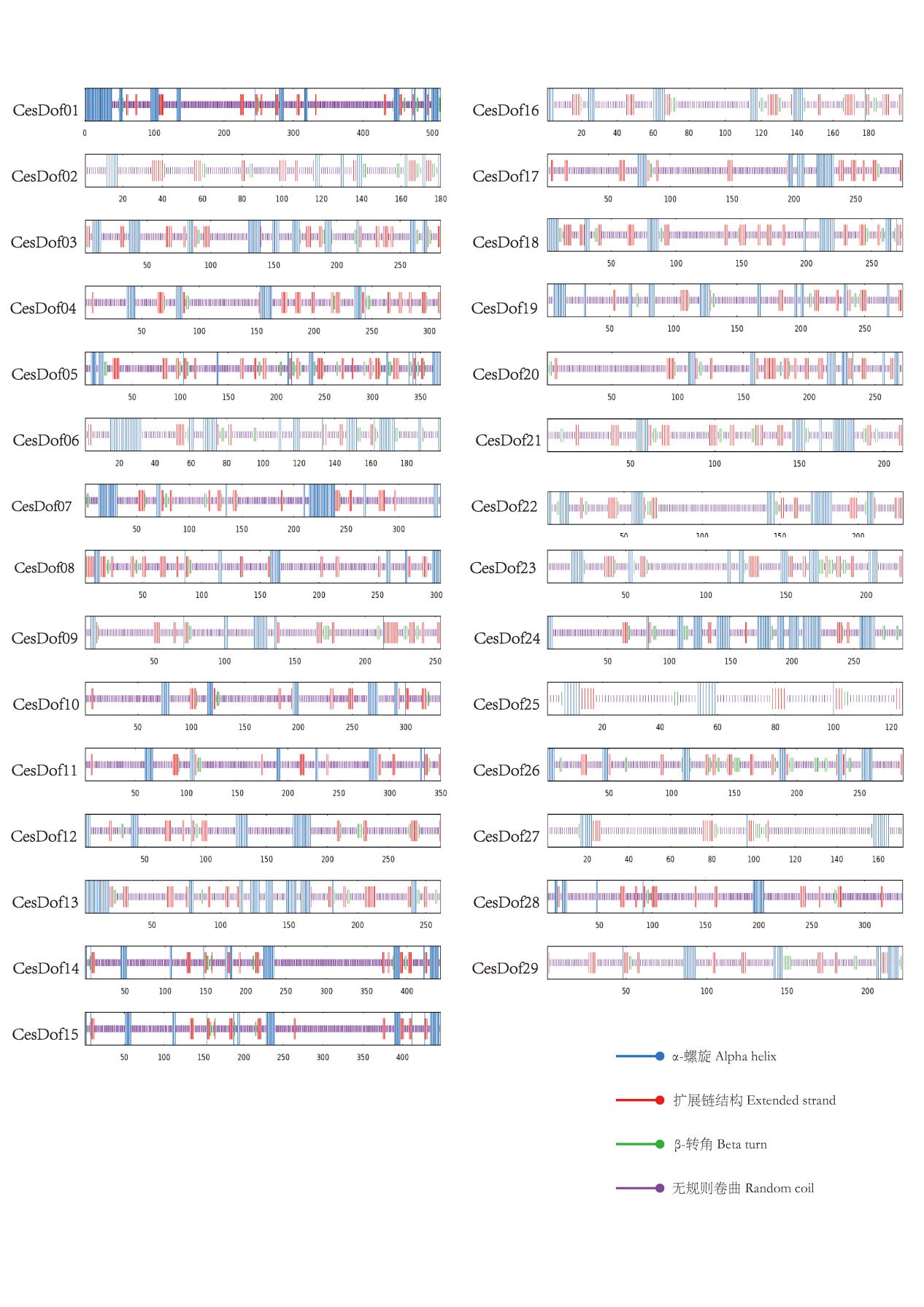


Supplementary Fig.1 Secondary structure of Dof proteins in *C.esculentus*


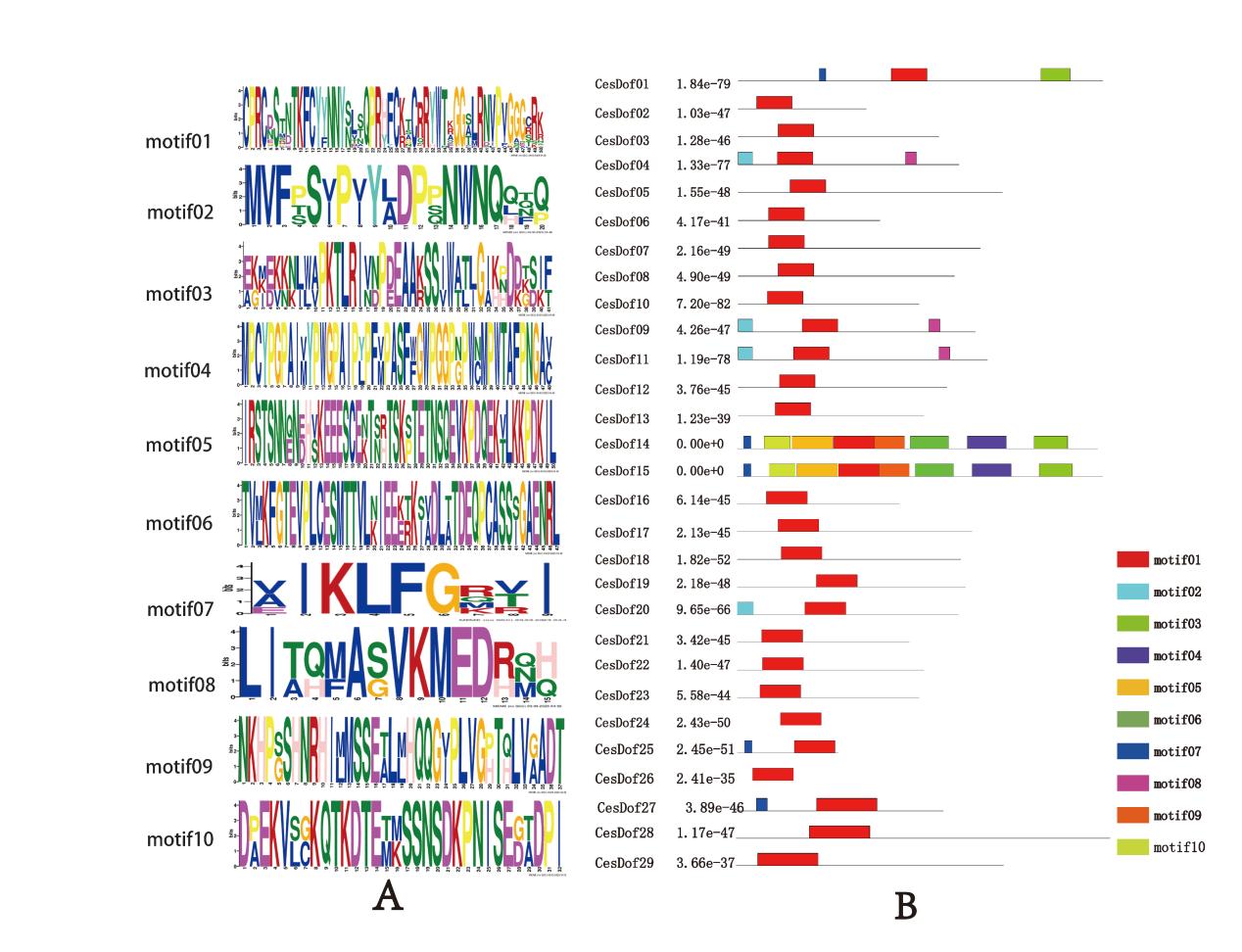


Supplementary Fig.2 Sequence logo of the conservative motifs of *Dof* family and conservative motif of family members in *Cyperus esculentu*
